# Reclaimed GFRP Sandwich Laminate from Decommissioned Wind Turbine Blades as a Road Noise Barrier Material: Full-Scale Acoustic Characterisation

**DOI:** 10.3390/ma19194106

**Published:** 2026-09-25

**Authors:** Filip Broniewicz, Mirosław Broniewicz, Elżbieta Broniewicz, Karolina Dec

**Affiliations:** Faculty of Civil Engineering and Environmental Sciences, Bialystok University of Technology, 15-351 Bialystok, Poland

**Keywords:** reused materials, wind turbine blade repurposing, acoustic performance, GFRP sandwich composite, road noise barrier, circularity, sustainable building materials, airborne sound insulation

## Abstract

Glass-fibre-reinforced polymer (GFRP) sandwich laminate recovered intact from decommissioned wind turbine blades is a candidate construction material: it is available in large panel sizes, requires no thermal or chemical processing, and has a documented structural performance. However, no acoustic characterisation of it has been reported. This study provides that characterisation for the road noise barrier application. A full-scale 4 × 4 m barrier panel was built from segments cut from decommissioned blades. A solid spar-cap laminate forms the lowest 1.2 m, and the sandwich shell forms the remainder, giving a mean surface mass of 39.6 kg/m^2^. Sound reflection and airborne sound insulation were measured in situ according to EN 1793-5 and 1793-6. The as-installed prototype reached *DL_SI,E_* (200–5000 Hz) = 29.6 ± 2.1 dB and *DL_RI_* = 0.1 ± 1.4 dB. It meets the 24–28 dB tender threshold most common among European road administrations, at one-fifth of the concrete’s surface mass. At 2000 Hz, positions facing the panel base fell 17.5 dB below their mass-based expectation, identifying a 5 cm mounting gap, not the material itself, as the origin of spatial variation. Away from the gap, per-band SIE reached 38–47 dB between 2500 and 5000 Hz, of the same order as values reported for purpose-made GFRP sandwich panels. These results form a prototype-level verification that a road noise barrier made of reclaimed blade laminate can meet the airborne sound insulation levels required in Europe.

## 1. Introduction

### 1.1. Wind Turbine Blade End-of-Life Challenge

Decommissioned wind turbine blades are a source of glass-fibre-reinforced polymer (GFRP) laminate that is available in large panel sizes, carries a documented structural history, and can be recovered without any thermal or chemical processing. The waste stream is growing: Europe’s wind fleet reached 304 GW of installed capacity at the end of 2025, and its earliest installations are now passing the end of their 20–25 year design life, so decommissioned blade material currently amounts to some 20,000 tonnes per year in Europe and is expected to reach 55,000 tonnes per year by 2030 [1]. Turbine blades are manufactured from fibre-reinforced composites—typically glass fibres embedded in a thermosetting epoxy matrix. While approximately 85–90% of a wind turbine’s mass (steel towers, concrete foundations, generators) can be recycled, the blades remain a major challenge. The cured thermosetting matrix cannot be remelted or separated into its original constituents. Existing processing methods, such as pyrolysis, co-incineration and mechanical grinding, are energy-intensive and give only downcycled products with reduced mechanical properties [2]. In many countries, recycling composites remains more expensive than landfilling, which encourages stockpiling. The blades are stored in warehouses or deposited in landfills, where they can persist for centuries with little structural change.

Recent regulatory developments are increasing pressure to find solutions. The European wind industry has committed to a self-imposed ban on landfilling blades starting January 2026 [1]. Polish regulations already classify composite blade waste as hazardous [3]. All these regulations create an urgent need for alternatives that go beyond downcycling.

Direct reuse of decommissioned blades is a more material-efficient alternative to recycling. Blades contain two laminate types: solid laminates in load-bearing areas and sandwich laminates in the aerodynamic shells. The sandwich shells consist of glass-fibre-reinforced polymer (GFRP) skins bonded to a balsa or foam core, with a single shell thickness of about 30 mm (24 mm core). Panel sections of usable size can be cut from the central span of the blade (approximately 3 to 23 m from the hub), where the curvature is sufficiently small for planar applications. The combination of GFRP skins and a lightweight core provides a high stiffness-to-weight ratio and surface densities one order of magnitude below those of concrete or masonry barriers, suggesting that the material warrants evaluation against the airborne sound insulation and reflection categories defined in the EN 1793 series.

Prior studies by the authors established the structural feasibility of this reuse path. Broniewicz et al. [4,5] characterised the mechanical properties of the GFRP laminates through material testing, developed a finite element model of the composite panel, and verified its load-bearing capacity under wind and traffic loads in accordance with European standards. A subsequent study extended this work to the conceptual design of the panel assembly and manufacturing process. Those investigations were confined to the structural domain. They did not measure the acoustic performance of the assembled panel and did not provide any acoustic properties.

Structural adequacy is a necessary but insufficient condition for deployment as a road noise barrier. A panel that withstands design loads must also achieve the airborne sound insulation and reflection ratings prescribed by the EN 1793 series. The acoustic performance of barrier panels made from reused wind turbine blade segments has not been reported, and the literature contains no data on such panels. The present study addresses this gap. It provides the first direct field acoustic evaluation of a full-scale prototype barrier constructed from end-of-life wind turbine blade segments, covering both sound reflection (EN 1793-5 [6]) and airborne sound insulation (EN 1793-6 [7]).

### 1.2. State of the Art

The role of noise barriers used in road engineering is to block noise generated by road traffic. This results in a lower noise level on the other side of the barrier, i.e., on the receiver side. Three acoustic mechanisms are at play: sound transmission through the barrier, sound reflection from the barrier surface, and diffraction of sound waves over the barrier’s top edge [8]. One of the most common materials (nearly 50% of all barriers in the USA) for noise barrier panels is reinforced concrete. Concrete barriers exhibit high sound insulation, at 40–50 dB. Their sound reflection is near-total (*DL_RI_* < 1 dB) when no surface treatment is applied, but absorptive textures can substantially reduce reflection [9].

There is an emerging trend in the use of recycled materials in acoustic applications. Most studies, however, concern the absorptive properties of porous materials [10] or address only the infill materials of barrier panels [11,12]. GFRP sandwich panels are used in construction, such as bridge decks [13] or cladding [14], but their acoustic properties have received limited attention. Studies [15] on GFRP composite sandwich floor panels show their airborne sound reduction value of 38 dB, which is acceptable for a road noise barrier. A research gap remains regarding the acoustic properties of reused sandwich composites as reflective/insulating barriers, and the authors are aware of no prior study that has investigated this or tested such panels.

Besides applications as barriers, the airborne sound insulation of sandwich panels has been studied mainly for construction elements, where the core has both an acoustic and structural role. Work on honeycomb and web-core panels has addressed the effect of core geometry, core filling and face damping on the transmission loss and on the resonance features of the spectrum [16,17]. That research deals with panels designed as acoustic elements, whereas in the present case the laminate architecture is inherited from an aerodynamic structure and cannot be modified.

Research published in the last five years has addressed two adjacent topics relevant to this work. The first is end-of-life wind turbine blade management. Recent reviews have catalogued mechanical, thermal and chemical recycling routes for GFRP blade material and quantified their energy and environmental costs [2,18]. The Re-Wind Network has demonstrated direct structural reuse of blade segments for pedestrian bridges [19,20]. This literature has not reported acoustic applications. The second topic is the acoustic performance of barriers and panels incorporating recycled or reclaimed materials. Recent work has characterised barriers and panels with recycled rubber granulate and tyre-derived aggregate [12] and recycled textile and cellulose fibres for absorptive layers [21]. None of these studies addresses the use of intact reclaimed GFRP sandwich laminates as the structural and acoustic element of the barrier itself. The present study fills this gap and provides the first EN 1793-5 and EN 1793-6 direct field evaluation of a full-scale prototype assembled from end-of-life wind turbine blade segments.

The acoustic performance of road noise barriers introduced to the European market is evaluated in accordance with the EN 1793 series of standards. The single-number sound reflection index *DL_RI_* and the single-number airborne sound insulation index *DL_SI,E_* are computed from the per-band reflection coefficient *RI* and the per-band sound insulation index *SI_E_* using the road traffic noise reference spectrum defined in EN 1793-3 [22].

Parts 1 and 2 of the series describe laboratory method testing; parts 5 [6] and 6 [7] regard in situ methods. The in situ methods, based on the Adrienne impulse response technique, were developed to address the gap between laboratory and field performance. In situ methods detect installation defects, such as air gaps, that laboratory tests miss [23]. Monitoring campaigns have applied the method to barriers in service [24].

### 1.3. Objective of the Study

This study evaluates, under direct field sound conditions, the acoustic performance of a full-scale road noise barrier prototype constructed from reused wind turbine blade segments, and establishes whether this end-of-life pathway produces a product that meets the standards of its intended market. The authors briefly summarise the prototype’s structural adequacy, as demonstrated in [4]. The measured data are analysed in terms of mass-law predictions, coincidence effects, and spatial variation across the panel. The nine positions of the EN 1793-6 grid serve as a diagnostic tool to separate the intrinsic performance of the reclaimed laminate from mounting effects, with particular attention to sound leakage at the panel’s base. To the authors’ knowledge, this is the first acoustic characterisation of a road noise barrier built from reused wind turbine blades, and the first to report per-position EN 1793-6 results for a full-scale orthotropic composite barrier. The measurements therefore establish, at prototype level, the acoustic performance of this reclaimed material as a building product, and supply the evidence required to assess reuse on the basis of what the recovered laminate yields rather than on the properties of the waste stream.

## 2. Materials and Panel Construction

The tested roadside noise barrier prototype features a 4 × 4 m composite acoustic panel mounted between supporting HEA steel posts. The internal panel frame is constructed from aluminium C-profiles. The top and bottom horizontal profiles span the full 4 m width of the barrier, while a central vertical post divides the frame into two distinct spans: one 1 m wide and the other 3 m wide. This central post remains visible on the exterior of the assembled panel. The developed manufacturing technology potentially enables noise barriers up to 6 m long and 8 m high, allowing for broad application in noise screen designs [5]. The acoustic panels can be used in road infrastructure for roadside screens, industrial acoustic cabins, enclosures, and barriers for machinery or other noise sources (Figure 1).

The acoustic panel is assembled from horizontal strips cut from the GFRP sandwich shells of decommissioned blades. These strips, measuring 1 m and 3 m in length to fit their respective spans, are stacked vertically to reach the total barrier height of 4 m. Most strips are 30 cm high, supplemented by 40 cm high strips to precisely match the target dimension. Specialised 3M™ tapes (Industrial Adhesives and Tapes Division 3M Poland, Nadarzyn, Poland), designed for bonding composites, are applied along the horizontal joints between the stacked strips. These tapes connect and stabilise the elements while minimising the acoustic leakage through gaps or slits between them. The strips forming the lowest 1.2 metres of the panel’s height are cut from the spar cap part of the wind turbine and are made of Type C laminate (Table 1). The spar caps occupy only about 18% of the shell perimeter but carry a substantial share of the blade’s mass, so a panel made only from sandwich strips would leave most of the blade’s mass unused. The rest of the panel utilises a double-wall design, meaning two independent layers of Type D sandwich strips are installed back-to-back without being physically joined through the panel thickness. At the horizontal edges where the two composite halves contact, the total solid thickness is 60 mm. At the mid-height of each segment, the inherent curvature of the blade strips opens a lens-shaped internal air cavity up to 30 mm, resulting in a maximum panel thickness of 90 mm.

The single-layer solid zone has a surface mass of 58.5 kg/m^2^, while the double-layer sandwich zone reaches 31.5 kg/m^2^. The panel’s calculated average surface mass is therefore 39.6 kg/m^2^. This is approximately one-fifth the surface mass of a typical concrete barrier panel (180–240 kg/m^2^), offering potential advantages in reduced foundation requirements and easier handling during installation. The mechanical properties of the individual laminates are presented in Table 1. The sandwich laminate core is balsa wood, which exhibits sound-absorbing properties [25], making it suitable for use as an acoustic panel in road noise barriers. At the same time, it maintains the mechanical strength required for use in standard roadside noise barriers.

The panel is therefore deliberately non-homogeneous, and this has two consequences for the acoustic analysis that follows. First, the mass-law reference against which the measured insulation is compared is not a single curve but one curve per zone (Section 5.1). Second, the nine microphone positions of the EN 1793-6 grid do not all face the same laminate, so the spatial variation of the measured sound insulation index cannot be attributed to material variability alone. Section 5.2 uses this to separate the recovered material’s intrinsic performance from the effect of mounting.

The barrier walls analysed in this study were assembled from segments cut exclusively from the mid-span region of multiple decommissioned blades; the root and tip sections were disregarded owing to unsuitable curvature. This limits but does not eliminate intra- and inter-segment variability: different blades differ in manufacturer, service history and lay-up tolerance, and within a single mid-span region, curvature, balsa-core density, and outer-face ply count can vary locally. Mechanical characterisation of representative coupons from the source blades, reported by the authors in [4,5], together with visual and geometric inspection of each segment prior to bonding, was used to confirm that all segments met the nominal Type D sandwich specification summarised in Table 1. Intra-panel mapping of local stiffness, thickness, or surface mass was not performed; the values in Table 1 and the derived bending stiffnesses used in Appendix A therefore represent specification averages rather than measured distributions across the prototype.

The tested prototype of the roadside barrier consists of a composite acoustic panel mounted between supporting posts made of HEA steel beams. The composite acoustic panel, 4000 mm high and approximately 4000 mm long, features a dual-layer design made of two composite walls cut from wind turbine blades, which serve as the panel’s external cladding. The barrier walls are constructed from uniform sheets cut from different sections of the blades, exhibiting locally varying curvature and internal structure. The panel is mounted on a 40-centimetre-high steel stand. There is a 5 cm gap between the top surface of the steel stand and the bottom of the aluminium frame (Figure 2).

Experimental tests on the full-scale acoustic panel were conducted using a dedicated testing stand. Maximum deflection ranged from 25 to 29 mm, with a limit of 50 mm. Inspection found no cracks or laminate delamination. Numerical analyses of the screen were also carried out using the finite element method. The stresses obtained from the FEM analysis were used to assess the panel’s load-bearing capacity according to the composite material failure criteria specified in the document [26]. The assessment was successful—the maximum composite stress reached 82% and was calculated for the outer laminate layer under the tensile failure criterion.

## 3. Acoustic Test Methodology

### 3.1. Test Standards

The acoustic performance of road traffic noise barriers in Europe is characterised according to the EN 1793 series of standards. The laboratory methods are described in EN 1793-1 (sound absorption under diffuse sound field conditions) and EN 1793-2 [27] (airborne sound insulation under diffuse sound field conditions). The more recently developed in situ methods are described in EN 1793-5 (sound reflection under direct sound field conditions) and EN 1793-6 (measurement of airborne sound insulation). For this study, in situ methods offer several advantages over laboratory testing. First, they allow evaluation of full-scale prototype barriers that may be too large for standard laboratory reverberant rooms. Second, they are sensitive to installation-specific features—such as gaps between panels, joint details, and connections to supporting posts. For these reasons, we applied the in situ methods described in EN 1793-5 and EN 1793-6 to assess the barrier’s acoustic properties.

The in situ methods are based on the Adrienne impulse response technique, in which a loudspeaker emits a deterministic excitation signal and the impulse response is derived from the recorded signal at the microphone positions. This technique was developed to address the problem that laboratory methods that test specimens under controlled conditions may not reflect installed performance. Garai and Guidorzi [28,29] show that by using the Adrienne method, one can obtain a good correlation between laboratory and in situ tests. Notable differences remain due to the sound field and, especially, mounting conditions. Arguably, the biggest disadvantage of the in situ method is that it requires skilled operators to be correctly applied, and measurement results can be sensitive to the operator’s experience and adherence to the prescribed procedure.

### 3.2. Sound Reflection Measurement (EN 1793-5)

The sound reflection coefficient was measured using the Adrienne measurement method. A controlled sound source (speaker) emits a transient sound wave that passes through the microphone position to the test device and then reflects off it (Figure 3). The microphone, positioned between the sound source and the test device, captures both the direct acoustic pressure wave travelling from the source to the device and the reflected (including scattered) acoustic pressure wave from the device. The power spectra of the direct and reflected components, corrected for the path length difference between them, form the basis for calculating the reflection coefficient. A speaker-microphone assembly was used, with the microphone rigidly mounted in front of the speaker at a distance of 1.25 m from it and 0.25 m from the reference plane of the noise barrier; this assembly can rotate both vertically and horizontally. The reference position is defined as the point where the microphone is located when the speaker-microphone assembly is set horizontally perpendicular to the tested noise barrier at mid-height of the barrier (Figure 3) and as far as possible from the sample edge. Around the reference position, a set of measurement positions was defined, including the reference position itself (Figure 4).

The sound reflection coefficient of road noise barriers was determined in accordance with the reference methodology of EN 1793-5. The expanded measurement uncertainty *U*_95_ was estimated according to Annex B of the standard, based on the standard deviation of reproducibility with a coverage factor *k* = 1.96 (corresponding to a 95% coverage probability). Prior to measurements, the measurement chain was calibrated, and deviations in the meter’s response to the calibration signal were determined before and after the measurements. Figure 5 shows the measurement setup. *DL_RI_* is the single-number, spectrum-weighted descriptor derived from the frequency-dependent reflection index *RI*; similarly, *DL_SI,E_* is derived from the frequency-dependent sound insulation index *SI_E_*.

### 3.3. Airborne Sound Insulation Measurement (EN 1793-6)

The sound insulation performance of a prototype acoustic screen built from panels made of recycled composite material from decommissioned wind turbine blades was measured in accordance with EN 1793-6. This standard defines the sound insulation index, which characterises a device’s capacity to limit sound transmission. The standard evaluates the sound insulation effectiveness of roadside noise-reducing devices (such as acoustic screens) under real operating conditions, within an acoustic field that is predominantly directional. The standard defines a measurement method for the airborne sound insulation of noise barriers installed along roads and allows the procedure to be applied both in the laboratory and in situ to existing structures.

Measurements were performed across the acoustic element only, so the global rating *DL_SI,G_* defined in clause 5.8.4 of [7] equals the element rating *DL_SI,E_* reported here. The test specimen measured 4 m in height by 4 m in length, less than the minimum dimension of 4 m by 6 m specified by the standard for qualification tests. Therefore, the rating reported is informative in the sense of clause 5.8.2 and is reported with its frequency range accordingly. This is a characterisation of a prototype rather than a qualification for a product.

Because of the sample dimensions and the design of the tested screen, measurements were carried out only on the panel, which represented the entire prototype structure prepared for testing. The expanded measurement uncertainty *U*_95_ was estimated in accordance with EN 1793-6, based on the reproducibility standard deviation (Clause B.3 of the standard) and using a coverage factor *k* = 1.96, corresponding to a 95% coverage probability for a normal distribution. The schematic layout of the measurement setup is shown in Figure 6, and views of the loudspeaker and microphones are shown in Figure 7.

### 3.4. Instrumentation and Signal Processing

Both the sound reflection and sound insulation measurements used the same instrumentation and signal-processing parameters (Table 2).

The expanded uncertainty *U*_95_ reported for the per-band sound insulation index *SI_E_* and the per-band sound reflection index *RI* was evaluated in accordance with Annex B of EN 1793-6 and EN 1793-5, respectively. For each one-third-octave band, the reproducibility standard deviation tabulated in the standard (EN 1793-6 Annex B, Table B.1 for sound insulation; EN 1793-5 Annex B, Table B.1 for sound reflection) was applied, and the expanded uncertainty was calculated as *U*_95_ = *k · sR* with coverage factor *k* = 1.96, corresponding to a coverage probability of approximately 95% assuming a normal distribution. Table 3 reports the single-number ratings of measurement results, Table 4 (sound reflection) and Table 5 (sound insulation) report the per-band uncertainties alongside the measurement results.

The single-number ratings *DL_SI,E_* and *DL_RI_* were computed from the per-band measured values using the normalised road traffic noise spectrum defined in EN 1793-3, in accordance with the recommended use of these indices for road traffic noise barriers. The laboratory-issued test reports provided per-band *SI_E_* and *RI* values, together with the corresponding expanded uncertainties. The authors recomputed the single-number ratings reported in this work from the accredited per-band values under the EN 1793-3 road traffic spectrum. The expanded uncertainty of the single-number ratings was estimated as *U*_95_ = *k · sR* with *k* = 1.96 from the reproducibility standard deviation tabulated in Annex B of the respective standards, giving *U*_95_ ≈ 2.1 dB for *DL_SI,E_* and *U*_95_ ≈ 1.4 dB for *DL_RI_*. These values are taken as approximate, since the tabulated *sR* is referenced to a different normalised spectrum.

The measurement chain is traceable to national standards through the accredited calibration certificates of the 1/2-inch condenser microphones (9 × Behringer ECM-8000, Behringer GmbH, Kirchardt, German), the sound calibrator (SONOPAN KA-50, PPHU SONOPAN Sp. z o.o., Białystok, Poland, certificate 1734/K/2024), the 18-channel audio interface (Behringer XR18 AIR, Behringer GmbH, Kirchardt, Germany, certificate 2423/2023) and the laser distance meter (Leica Disto D510, Leica Geosystems AG, Heerbrugg, Switzerland, certificate 3784/AM/23), all issued by calibration laboratories accredited by the Polish Centre for Accreditation (PCA), signatory of the EA MLA and ILAC MRA. Repeatability of the measurement chain was verified by comparing the response to the calibration signal (94.0 dB) before and after the measurement campaign. The maximum drift across all nine channels was 0.2 dB, which is within the laboratory acceptance threshold of 0.5 dB. Systematic effects that the Annex B uncertainty does not resolve as separate components, namely source directivity, microphone-array positioning tolerance, and propagation-path corrections for temperature, humidity and atmospheric pressure, were handled at the measurement stage through the frequency-dependent directivity correction *C_dir_* and gain correction *C_gain_* applied during post-processing as prescribed by the standards. Environmental conditions during the measurement campaign were stable (15 °C, 53% relative humidity, 1018.7 hPa, wind speed 0.0 m/s).

At *f_s_* = 48 kHz, the *N* = 32,768 sample MLS sequence has a duration of *T* = *N/fs* ≈ 0.683 s and a theoretical spectral resolution of *Δf* = *fs/N* ≈ 1.46 Hz. This is below the 46 Hz bandwidth of the lowest evaluated one-third octave band at 200 Hz. The 200 Hz lower limit is therefore not set by the MLS parameters but by the Adrienne temporal window, whose length must be short enough to exclude the first parasitic reflections from the impulse response. The window lengths of 7.9 ms (positions 1–6) and 5.9 ms (positions 7–9, closer to the ground/gap) were selected on that basis and are consistent with the 200 Hz floor fixed by EN 1793-5 and 1793-6. The 32,768-sample sequence, therefore, exceeds what is strictly required for spectral resolution at 200 Hz. The minimum characteristic dimension of the test sample space was 4.0 m. Figure 8 shows the microphone array configuration.

The single-number ratings were computed according to Formula (8) of [7], in which the summation starts at the lowest reliable one-third octave band. In this case (7.9 ms window, positions 1–6) this band is 200 Hz, so the values measured in the 100, 125 and 160 Hz bands are reported in Table 5 for information only. The rating is therefore *DL_SI,E_* (200–5000 Hz). For positions 7–9 the lowest reliable band is 250 Hz. The restriction of the summation to 250–5000 Hz changes the rating from 29.6 dB to 29.8 dB, therefore the choice of lower limit does not affect the conclusions.

## 4. Results

### 4.1. Sound Reflection Results (EN 1793-5)

Table 3 presents the single-number rating *DL_RI_* obtained for the barrier; Table 4 lists the underlying sound reflection index (*RI*) values for third-octave frequency bands, as per EN 1793-5. The accredited test report issued by the laboratory provided the per-band *RI* values together with their band-wise expanded uncertainties. The authors computed the single-number rating reported here from those accredited per-band values using the normalised road traffic noise spectrum defined in EN 1793-3 [22], in accordance with the recommended use of *DL_RI_* for road traffic noise barriers. The evaluation follows EN 1793-5 [6].

The chart shown in Figure 9 illustrates the sound reflection coefficient (*RI*) for one-third octave frequency bands, as defined in EN 1793-5. In the standardised evaluation range (200–5000 Hz), the position-averaged *RI* oscillates between 0.74 and 1.11. Most values cluster around 0.91–1.03, close to the reference value of 1.0 expected for a dense, stiff, non-perforated panel with high surface impedance. The single-position values reach 1.30 at 2500 Hz (position 1) and exceed 1.0 in eight bands between 250 and 4000 Hz, with the position-averaged *RI* exceeding 1.0 in four bands (1600–3150 Hz).

Values above unity are within the expanded uncertainty *U*_95_ = 0.20–0.27 of the physical upper bound *RI* = 1.0 and are a measurement artefact of the EN 1793-5 method, attributable to edge diffraction from the finite-size sample and reflections from the panel mounting frame, both of which fall inside the Adrienne analysis window. This does not, however, affect the single-number rating. Clipping every per-band value to the physical bound *RI* = 1.0 before applying the normalised spectrum changes *DL_RI_* from 0.13 to 0.18 dB, a difference of 0.05 dB against an expanded uncertainty *U*_95_ of 1.4 dB. The reported value of 0.1 dB is unaffected at the precision quoted. The reduction at 5000 Hz (*RI* = 0.74, *U*_95_ = 0.37) reflects the lower signal-to-noise ratio at the upper edge of the source bandwidth. The largest scatter between positions occurs at 2500 and 5000 Hz, consistent with the panel’s curved surface geometry.

### 4.2. Sound Insulation Results (EN 1793-6)

The accredited test report provided the per-band sound insulation index (*SI_E_*) values together with their band-wise expanded uncertainties. The authors computed the single-number rating from these per-band values using the normalised road traffic noise spectrum defined in EN 1793-3, in accordance with the recommended use of *DL_SI,E_* for road traffic noise barriers. The rating of airborne sound insulation derived in this way is *DL_SI,E_* = 29.6 ± 2.1 dB, which exceeds the minimum value typically required for highway noise barriers. Table 5 presents the per-band *SI_E_* values measured in accordance with EN 1793-6. Figure 10 illustrates the *SI_E_* for one-third octave frequency bands, as defined in EN 1793-6.

The values show a general upward trend with frequency: the logarithmic average rises from 24.4 dB (200 Hz) to 39.1 dB (4000 Hz). The overall *SI_E_* increase across the 200–5000 Hz range is approximately 15 dB. The specific features of the curve are easily visible in Figure 10. A small dip occurs at 500–800 Hz, and a more pronounced one at 2000 Hz with a magnitude of approximately 5.5 dB. The curve peaks at 4000 Hz (39 dB), followed by a slight drop at 5000 Hz.

The ranges of particular values per position are: *SI*_1_—19.1–46.3 dB, *SI*_2_—24.2–45.5 dB, *SI*_3_—30.1–46.8 dB, *SI*_4_—21.3–41.8 dB, *SI*_5_—26.1–47.5 dB, *SI*_6_—28.1–45.8 dB, *SI*_7_—21.8–34.8 dB, *SI*_8_—26.2–39.1 dB, *SI*_9_—25.9–42.8 dB. Some spatial variation patterns can be noticed. Positions 1–3 generally exhibit higher *SI_E_*, while positions 7–9 exhibit lower ones. There are some frequencies at which the spatial variation between positions is very noticeable. At 2000 Hz, the difference between *SI*_3_ and *SI*_7_ is 17.9 dB, while at 630 Hz, the difference between *SI*_2_ and *SI*_9_ is nearly 10 dB.

## 5. Discussion

### 5.1. Acoustic Performance of the Panel

The measured *DL_RI_* = 0.1 dB indicates near-complete sound reflection with no absorption, as expected for a non-perforated panel of high surface impedance. Treating each zone as a limp mass, the surface impedance is:

(1) $$Z_{s}=j\cdot \omega \cdot m_{s},$$

where *j* is the imaginary unit, surface mass *m_s_* = 31.5 kg/m^2^ or 58.5 kg/m^2^, and *ω* = 2π*·f*. At a representative frequency of 1 kHz, this gives *Z_s_* ≈ 198,000 or 367,500 Pa·s/m, approximately 477 times (for the sandwich zone) or 886 times (for the solid zone) the characteristic impedance of air (ρc ≈ 415 Pa·s/m). This ratio increases linearly with frequency, so the impedance mismatch is even larger at higher frequencies. The lumped-mass approximation ignores orthotropic bending stiffness and core shear compliance, which reduce the effective impedance at and above coincidence (590–619 Hz, Appendix A). The mismatch is nevertheless large enough that virtually all incident energy is reflected across the measurement range.

This result is of the same order as typical values for plain concrete barriers without absorptive treatment, although their mass is much higher (180–240 kg/m^2^). The high *RI* > 1.0 values at some frequencies are a known measurement artefact of the EN 1793-5 method, possibly caused by diffraction at panel edges and scattering from the non-planar surface. These phenomena can concentrate energy towards the microphone, as documented in the literature [29].

The panel comprises two zones of different surface mass, so the mass-law reference against which the measurement is interpreted is not a single curve. We therefore computed normal-incidence transmission loss separately for 31.5 kg/m^2^ and 58.5 kg/m^2^. For normal-incidence sound, the theoretical transmission loss is given by:

(2) $$TL=20\cdot \log_{10}\frac{\pi \cdot f\cdot m_{s}}{\rho \cdot c},$$

where *m_s_* is the surface mass of the panel, ρ = 1.21 kg/m^3^ and *c* = 343 m/s. This idealised formula assumes an infinite, perfectly limp mass (zero bending stiffness) subjected to a normal-incidence plane wave. In contrast, *SI_E_* is obtained from the Adrienne impulse-response method, which uses a finite panel and a sound source emitting spherical waves. The laminate also has substantial orthotropic bending and core shear stiffness. The theoretical TL values therefore represent an idealised baseline rather than a direct equivalent to the measured *SI_E_*.

The comparison in Figure 11 is therefore qualitative, highlighting where the measured spectrum deviates from the theoretical mass-controlled trend. Each zone is shown against its own reference curve. In both zones, the measured insulation lies below the corresponding prediction and rises more slowly than the mass-law prediction of 6 dB per octave across the 200–5000 Hz range. Above 1 kHz the curve for the heavier zone lies below that for the lighter one, the reverse of the mass-law ordering; Section 5.2 attributes this to the base gap. Two mechanisms account for the deviation: a coincidence effect near the lower end of the range (treated below) and acoustic leakage through the 5 cm gap at the base of the panel (Section 5.2).

Part of the deviation is due to coincidence. Applying thin-plate theory to each laminate separately gives critical frequencies of 590–619 Hz for the sandwich zone and 590–1162 Hz for the solid cap zone. Appendix A gives the derivation and the underlying stiffness values. Both zones reach the lower edge of their coincidence region in the same 590–620 Hz band. That edge is where the measured insulation first departs from the mass-controlled trend, and it is consistent with the plateau observed across the 500–800 Hz one-third octave bands (Figure 10). The plateau is present in all three position groups (Figure 12) even though the lowest group faces a different laminate from the other two. The two laminates reach coincidence at the same frequency by different routes. In the sandwich, the bending stiffness comes from the separation of thin, low-modulus faces. In the cap laminate it comes from the high modulus of a thicker solid section. The zones differ in how far the coincidence region extends upward. The sandwich is nearly isotropic in bending, so its coincidence stays within 590–619 Hz. The cap laminate has a stiffness ratio of about four between its two directions and spreads coincidence over 590–1162 Hz. A defect localised to one part of the panel would not reproduce this pattern, but a coincidence effect common to both laminates does.

The dip near 2000 Hz behaves differently. It is absent from positions 1–3, present in positions 4–6 and most pronounced in positions 7–9, deepening with proximity to the base of the panel. No coincidence mechanism produces an effect that is frequency-selective and at the same time varies systematically with position on a panel that is uniform within each zone. Section 5.2 examines this dip.

### 5.2. Installation Quality as the Origin of Spatial Variation

Positions 7–9 of the microphone grid are located approximately 0.7 m above the panel base and face the solid cap zone described in Section 2. The surface mass of this zone, 58.5 kg/m^2^, is 1.86 times that of the sandwich zone faced by positions 1–6. In the mass-controlled frequency region, this ratio corresponds to a difference of 5.4 dB. Based on surface mass alone, positions 7–9 would therefore be expected to yield the highest sound insulation index of the grid.

The measured values show the opposite trend. At 2000 Hz, the logarithmic average of positions 1–3 is 36.7 dB, which gives an expected value of 42.1 dB for positions 7–9. The measured value is 24.6 dB, a deficit of 17.5 dB with respect to the surface mass of the material at that location. Expressed as a difference between individual positions, *SI*_3_ exceeds *SI*_7_ by 17.9 dB in the same band. The mean deficit over the 1000–5000 Hz range is 13.6 dB. A deficit of this sign cannot be explained by the mechanical properties of either laminate, and its magnitude increases towards the base of the panel. The effect is attributed to sound leakage through the approximately 5 cm gap between the steel stand and the panel frame (Figure 13).

The frequency dependence supports this attribution. The deficit is 2.0 dB at 200 Hz, where the wavelength of 1.7 m exceeds the gap width by a factor of about thirty. It reaches up to 17.9 dB in the 2000–3150 Hz bands, where the wavelength is 0.11–0.17 m. The gap area, 0.05 × 4.0 = 0.2 m^2^, is 1.25% of the panel area, which for a partition in a diffuse sound field would limit the transmission loss to approximately 19 dB. The measured *DL_SI,E_* = 29.6 dB is higher because it is a nine-position average obtained in a direct sound field, in which only the lowest row samples the aperture. At those positions, the diffuse-field limit is approached, with *SI*_7_ = 21.8 dB at 2000 Hz.

A cross-section of the full assembly is shown in Figure 14. The double-wall arrangement leaves a lens-shaped air cavity of up to 30 mm at mid-height of each strip, and cavity resonance was considered as an alternative or contributing mechanism for the 2 kHz dip. Three estimates place it outside the relevant region. The mass–air–mass resonance of the two 15.8 kg/m^2^ walls lies at 123 Hz for a 30 mm separation and rises to 302 Hz where the cavity narrows to 5 mm, that is, at or below the bottom of the evaluation range. The first cross-cavity standing wave, at a half-wavelength of 30 mm, lies at 5.7 kHz, above it. Acoustic modes along the 0.3 m height of a strip fall at 572, 1143, 1715 and 2287 Hz and none of them coincide with the observed dip. The decisive argument is geometric: the dip is absent at positions 1–3 and most pronounced at positions 7–9, and positions 7–9 face the single-layer solid spar cap zone, which contains no cavity at all. A cavity mechanism would produce the opposite spatial distribution. The cavity is therefore not responsible for the dip.

Other paths may contribute. The taped joints between strips, the strip-to-frame connections and local gaps at segment interfaces are all candidates, but none is confined to the base, and none accounts for the vertical gradient, so the base gap is considered dominant. The result separates the intrinsic performance of the reclaimed laminate from that of the mounted assembly. Positions 1–3 reach per-band *SI_E_* values of 38–47 dB above 2000 Hz, and the material faced by positions 7–9 still has a higher surface mass. Laboratory testing of an isolated specimen would not reproduce the mounting condition and would not detect the effect. For products manufactured from reclaimed feedstock of variable provenance, this allows the performance of the recovered material to be assessed independently of installation quality.

The attribution of the deficit to the base gap is an inference drawn from four independent features of the dataset. First, the deficit scales with frequency in the manner expected of an aperture: it is 2.0 dB at 200 Hz, where the wavelength exceeds the gap width by a factor of about thirty, and reaches 17.5–17.9 dB in the 2000 and 3150 Hz bands, where the wavelength is comparable with it. Second, it increases monotonically towards the base of the panel. Third, it has the opposite sign to the mass-law expectation, since positions 7–9 face the heavier of the two zones. Fourth, no material mechanism is available that is frequency-selective and at the same time varies systematically with position within a zone that is uniform. This phenomenon is expected by the test method: clause 5.6.3 of EN 1793-6:2025 provides for a dedicated set of nine measurements taken with the grid placed close to a suspected leak, with the Adrienne window enlarged to admit the leaked signal, and states that in such a measurement the signal arriving from the bottom edge is the transmitted signal being sought rather than a parasitic one. That dedicated measurement was not part of the present study, in which the window at positions 7–9 was shortened to 5.9 ms to exclude late arrivals. The deficit reported here is therefore a leak detected within a standard element measurement.

### 5.3. Performance in Relation to Barrier Product Requirements

Table 6 compares the measured ratings with typical values reported for conventional barrier materials. The *DL_SI,E_* = 29.6 dB measured here falls within the range reported for concrete, metal and transparent barriers, and above the lower part of the range for timber. The comparison is made for in situ values. The spread within each class is wide, 14 dB for concrete and 20 dB for metal, and is not explained by surface mass, which varies by less than a factor of two within each class. What separates a barrier at the top of its class from one at the bottom is the sealing of joints, the post interface and the base detail, an effect quantified for the present panel in Section 5.2.

The only directly comparable measurement on a GFRP sandwich is that of Proença et al. [15], who reported an airborne sound reduction index of 38 dB for purpose-made GFRP-PUR web-core panels under laboratory conditions. The per-band SI_E_ of 38–47 dB reached here at positions away from the base gap is of the same order as that value, but the comparison is qualitative: the two panels differ in core material and thickness and [15] reports a laboratory diffuse-field index, which for a given element is generally higher than the in situ direct-field value [28]. It can be stated that two decades of service on an operating turbine, followed by sectioning and reassembly, do not place the reclaimed laminate in an evidently different performance range from purpose-made GFRP sandwich. Testing both by the same method is required to establish equivalence.

Whether 29.6 dB is adequate is better answered against what road authorities require than against the highest value attainable. A European survey covering 21 road and 6 rail administrations in 18 countries found the most frequently specified tender threshold for *DL_SI,E_* to lie between 24 and 28 dB, with 25 dB in Austria and Sweden, 28 dB in Belgium and France, and, in Italy, above 27 dB for the element together with above 24 dB for the post [32]. The classes B1 to B4 invoked by EN 14388 [34] are defined in EN 1793-2 [27] on the laboratory index *DL_R_*, whereas the present result is the in situ *DL_SI,E_*, for which [7] no longer permits categories of the single-number rating to be used. No classification is therefore claimed here. The measured 29.6 dB is, however, directly comparable with the tender thresholds cited above, which are specified in terms of in situ values, and exceeds the 24–28 dB range most frequently required. The prototype therefore satisfies the most common European requirement.

The threshold exists because once the transmission loss of the panel exceeds 25–30 dB, the insertion loss of the installed barrier is limited by diffraction over its upper edge rather than by transmission through its face, and further surface mass yields no corresponding gain in the field. The effect of the base gap on the rating can be bounded from the present dataset. Recomputing *DL_SI,E_* from the three positions furthest from the gap (Positions 1–3) gives 30.9 dB, against 29.6 dB for the full nine-position average and 28.5 dB for positions 7–9. Sealing the gap would therefore raise the rating by about 1 dB. The effect is small because the rating is governed by the lower part of the evaluation range, where the three rows of the grid give similar averages, and not by the 2 kHz region, where the difference between the rows is largest.

The rating is achieved at a mean surface mass of 39.6 kg/m^2^, approximately one-fifth of the mass of concrete barriers (180–240 kg/m^2^). The consequences are structural rather than acoustic: lighter panels reduce the foundation demand and can be handled with lighter equipment. Within the double-wall arrangement, the mass is distributed rather than uniform, with the heavier solid zone placed where the bending demand is greatest, so the reduction is obtained without a corresponding reduction in capacity.

The insulation rating does not capture two service-relevant properties. The first is durability, on which the comparison is least settled: timber barriers have been reported to lose 6–7 dB within a year of exposure [24], while the laminate used here spent more than two decades on operating turbines, which establishes that the material withstands prolonged outdoor exposure in its original form but not that the barrier will. We did not measure the residual mechanical and acoustic properties of reclaimed segments after a second exposure period; this is identified as future work. The second is reflection: the measured *DL_RI_* = 0.1 dB indicates near-complete reflection without surface treatment, placing the panel with untreated concrete. Reflection could be reduced by perforating the outer shell over an absorptive cavity, or by surface-mounted absorptive or vegetated layers, at the cost of the simplicity that makes the route attractive.

### 5.4. Material Supply from a Decommissioned Wind Farm

The supply available from a decommissioning project follows from the geometry of the recovered strips. The reference blade considered here is 25 m long, which corresponds to a turbine of roughly 600–850 kW, and yields 63.3 m^2^ of recoverable strip area. A 4 m high panel consumes 1.70 m^2^ of blade area for every 1 m^2^ of screen, so the area balance gives 37.2 m^2^ of finished barrier per blade. For a farm of 20 turbines with three blades each, this is about 2230 m^2^ of screen, equivalent to about 558 m of a 4 m high barrier. A single farm therefore supplies a measurable local material stream for specific road and rail investments rather than a solution at market scale.

The limit on this yield is the geometry of the blade, not the processing. Only the mid-span forms relatively flat strips. In the geometry adopted here, the useful window runs from about 3 m to about 23 m from the root, between the end of the root transition and the start of the slender, strongly twisted tip. Neither the root nor the tip can be used for flat screens. The remaining fractions, the root, the tip, the trailing edges, the shear webs and the cutting waste, have to be managed separately.

### 5.5. Implications for Material Supply and Circularity

The point at which the blade is sectioned is a decommissioning decision rather than a manufacturing one, and it has to be taken before the turbine is dismantled. Two options are available. In the first, the blade is lowered intact and moved whole to the processing site. This requires abnormal-load transport, route surveys, escort vehicles and permits, and allows one blade per journey. In the second, the root transition is removed at the turbine, and the remaining span is cut into segments of approximately 4 m, which travel on standard flatbed transport at three to eight segments per journey. The choice determines the site equipment, the work sequence, the containment measures required for cutting dust and fibre, and the form of the transport contract. It therefore belongs in the decommissioning plan rather than in a subsequent processing agreement. Sectioning should also be set out to preserve the productive span identified in Section 5.4, rather than dividing the blade into equal lengths from the root.

The barrier panel will itself reach end of life, and its disposal route depends on how it was assembled. The panel is a bolted aluminium frame carrying composite strips joined by structural adhesive tape, with no through-thickness bonding between the two walls and no resin infusion at any stage of manufacture. It can therefore be dismantled into the fractions from which it was built. The aluminium profiles and steel fixings are recoverable through established metal recycling routes. The composite strips return to the same set of options that the blade faced, in the same physical form and with the laminate architecture intact. Whether the mechanical and acoustic properties of the strips after a further service period would support a third use, or only the treatment routes available to blade material today, was not investigated here and is identified in Section 5.6 as a subject for further work.

More generally, the result addresses a gap in the way end-of-life options for blades are assessed. Repurposing routes are commonly evaluated on material recovery, processing energy or cost, all of which are properties of the route. None of these establishes whether the resulting object can be placed on its intended market, which is governed by the product standards of that market rather than by the properties of the waste stream. For road noise barriers, the relevant framework is the harmonised product standard EN 14388 together with the EN 1793 series, and the evidence required is a measured single-number rating obtained on a full-scale assembly under representative mounting conditions. Until that evidence exists, a repurposing route cannot enter an operator’s end-of-life decision on the same footing as recycling or energy recovery, for which performance data are established. The measurements reported here supply it for one route and one product. The same requirement applies to the pedestrian bridges, facade elements and retaining structures proposed elsewhere in the literature.

### 5.6. Limitations and Further Work

Four limitations bound the result. First, it is obtained on a single prototype assembled from segments of a small number of source blades, and on a specimen of 4 m by 4 m, below the 4 m by 6 m that clause 5.5.7 of [7] specifies for qualification tests. Therefore, the rating is informative in the sense of clause 5.8.2 of that standard, and characterises this configuration, not the waste stream. Second, intra- and inter-panel variability in curvature, core density and lay-up, inherent to a prototype assembled from multiple mid-span segments, was not characterised at the panel scale, and the analysis in Section 5.1 and Section 5.2 uses nominal properties. Its effect on the rating is bounded by the mass law: a ±10% spread in surface mass shifts the transmission loss by about ±0.9 dB and a ±20% spread by about ±1.9 dB, against an expanded uncertainty *U*_95_ of 2.1 dB on the rating itself. The 5.6 dB margin over the 24 dB tender threshold would require the surface mass to fall to 53% of nominal, outside any plausible spread, whereas the 1.6 dB margin over the 28 dB threshold could be consumed by a ±20% variation; panels intended for the most demanding specifications would therefore need either controlled selection of source segments or a verification measurement. Band-level features, notably the position of the coincidence region, are more sensitive to this variability than the rating is (Appendix A, Table A2). Third, the measurement applies to one zone proportion, 1.2 m of solid laminate beneath 2.8 m of sandwich; because the two zones differ in surface mass by a factor of 1.86, a panel built to a different split would not be expected to reach the same rating without a repeated measurement. Fourth, the supply figures in Section 5.4 are estimates based on a single reference blade and a single panel geometry.

Four items follow for further work. The principal one is confirmation of the rating on panels built from blades of different manufacturers, ratings and service histories, which is what would convert the present prototype-level result into a performance level for the stream. The second is a direct characterisation of the base leak, for which the test method itself provides the procedure: clause 5.6.3 of [7] prescribes an additional nine-position measurement with the grid placed close to the bottom edge and the Adrienne window enlarged accordingly. That measurement, together with a repeat of the element measurement with the gap sealed, would replace the inference of Section 5.2 with a direct result and would test the estimate of Section 5.3 that the gain in the rating is of the order of 1 dB; neither was possible within the present campaign. The third is the residual mechanical and acoustic performance of the reclaimed laminate after service as a barrier, on which the durability of a second-life route ultimately depends, and which cannot be inferred from the two decades the material spent on the turbine. The fourth concerns the acoustic treatment of the panel. Filling the inter-wall cavity with a lightweight damping medium has been reported to raise the mid- and high-frequency insulation of sandwich and honeycomb structures and to suppress resonance dips [16]. In the present panel the cavity arises from the natural curvature of the strips, so filling it would add a process step but leave the recovered material unchanged. Reducing the reflection index would require absorptive treatment of the outer shell, by perforation or by a surface-mounted layer as outlined in Section 5.3, which was outside the scope of the present prototype.

## 6. Conclusions

This study reports the first direct-sound-field acoustic characterisation of a full-scale noise barrier built from intact reused GFRP sandwich segments cut from decommissioned wind turbine blades. We measured sound reflection and airborne sound insulation according to EN 1793-5 and EN 1793-6. The main findings are as follows:

1. The single-number ratings were *DL_SI,E_* = 29.6 ± 2.1 dB and *DL_RI_* = 0.1 ± 1.4 dB, obtained at a mean surface mass of 39.6 kg/m^2^, approximately one fifth of that of concrete barriers. The rating exceeds the tender threshold of 24–28 dB most frequently specified by European road administrations. It is an informative rating in the sense of clause 5.8.2 of EN 1793-6:2025, the specimen being smaller than a qualification test requires, and no performance class is assigned to it.
2. The plateau across the 500–800 Hz one-third octave bands is a coincidence effect common to both laminates. Thin-plate theory places the lower edge of the coincidence region at 590–620 Hz in both zones, although the sandwich and the solid spar cap reach it through different cross-sections. The shear-corrected value for the sandwich zone is bounded only within 620–1190 Hz by the available balsa data, so the lower edge is the part of the prediction that the measurement resolves.
3. The dip at 2000 Hz is attributed to the mounting. Positions 7–9 fall 17.5 dB below the value expected from the surface mass at their location, and *SI_7_* is 17.9 dB below *SI_3_* in the same band. The deficit has the opposite sign to the mass-law prediction, which points to the 5 cm gap at the base rather than the laminate as the origin of the spatial variation of *SI_E_* above 1 kHz. Its effect on the single-number rating is about 1 dB. Positions 1–3 reach per-band *SI_E_* values of 38–47 dB in the 2500–5000 Hz range, so the reclaimed laminate supports higher insulation than the nine-position average indicates.
4. A farm of 20 turbines yields about 2230 m^2^ of screen, equivalent to about 558 m of a 4 m high barrier. The constraint is the blade geometry rather than processing. Only the span between about 3 and 23 m from the root forms usable flat strips.
5. Placing a repurposing route alongside recycling or energy recovery in an end-of-life decision requires evidence that the resulting product complies with the standards of its own market. For road noise barriers, that means EN 14388 together with a measured single-number rating obtained on a full-scale assembly. This study supplies that evidence for one route and one product. Extending it to a declared performance level would require measurements on a representative population of panels.

## Figures and Tables

**Figure 1:**
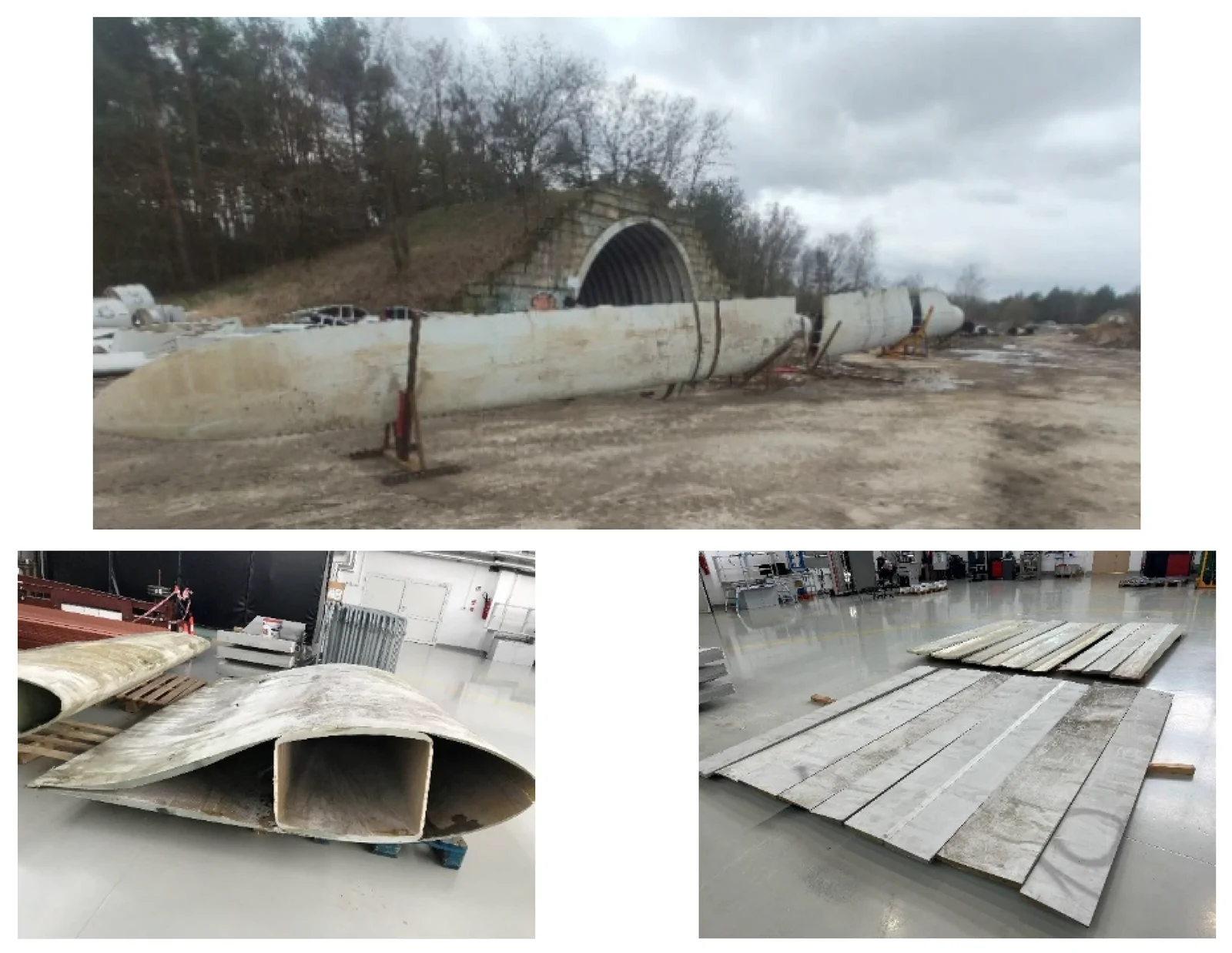
Source material and panel preparation: decommissioned wind turbine blade (**top**), 3 m longitudinal segment cut from the blade (**bottom left**), flat rectangular panels obtained from the segment, ready for frame assembly (**bottom right**).

**Figure 2:**
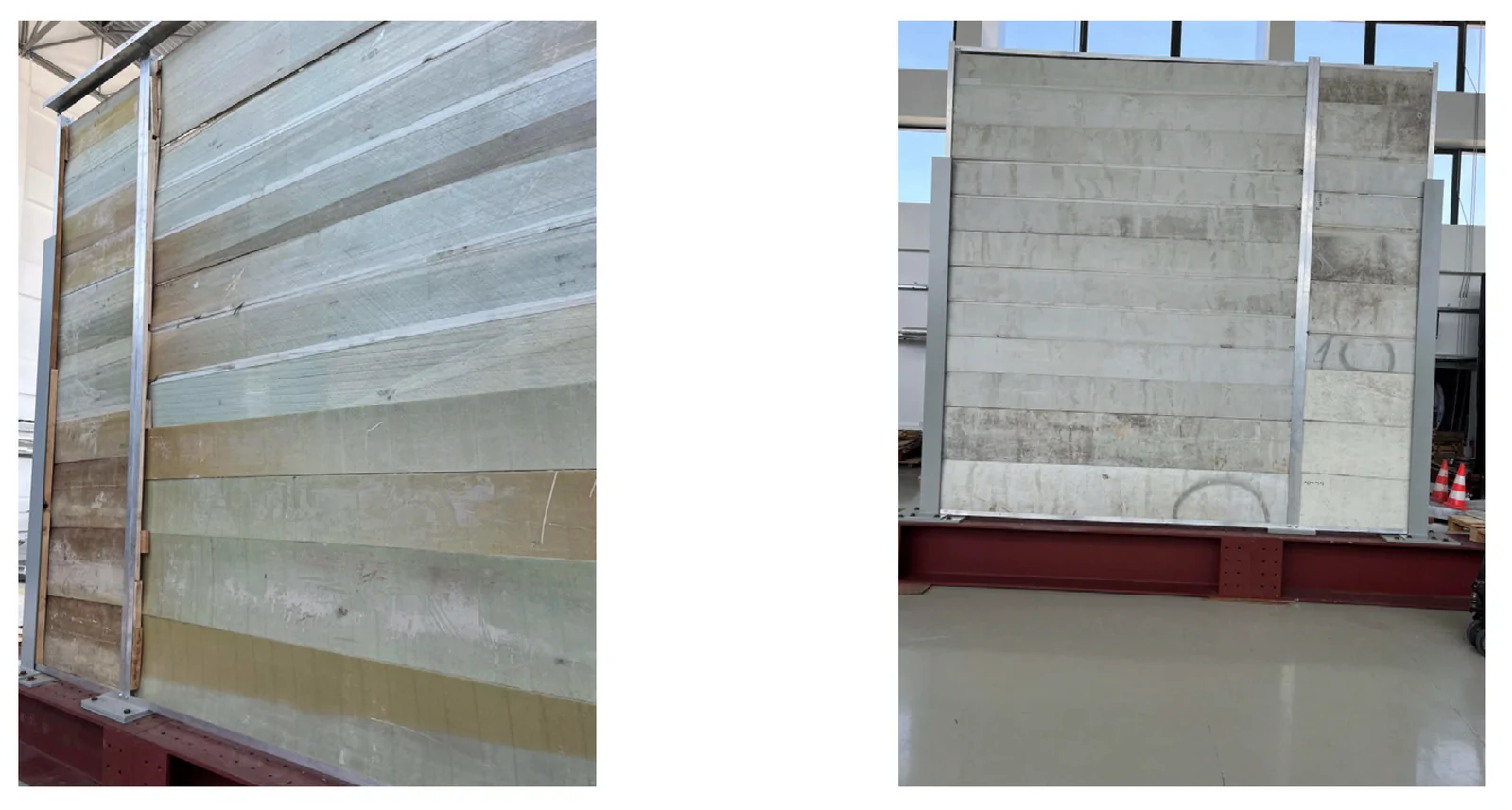
Panel screen fabricated from wind turbine blade composite materials investigated in this study.

**Figure 3:**
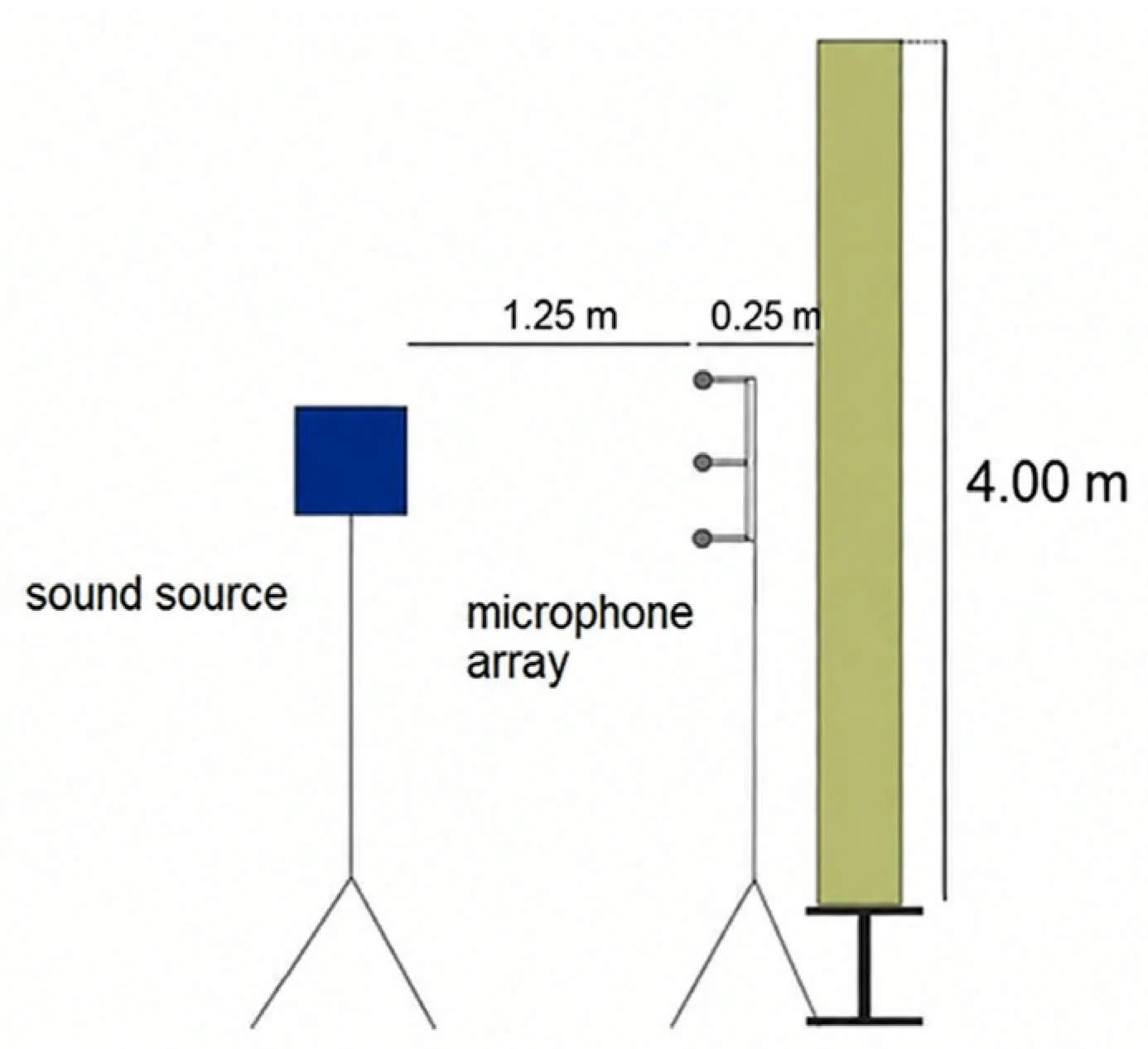
Side view of the sound reflection measurement system.

**Figure 4:**
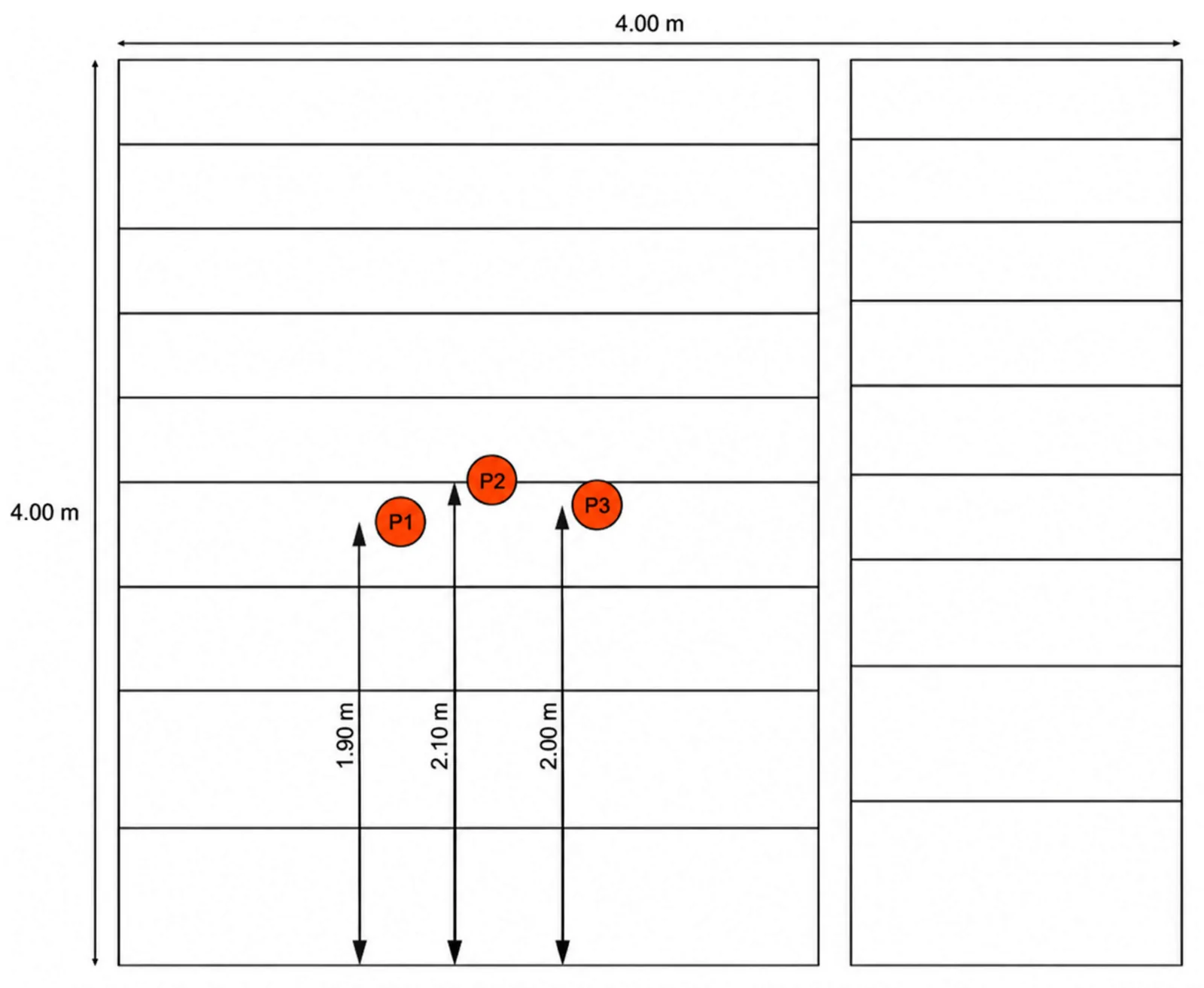
Measurement setup—front view of the screen.

**Figure 5:**
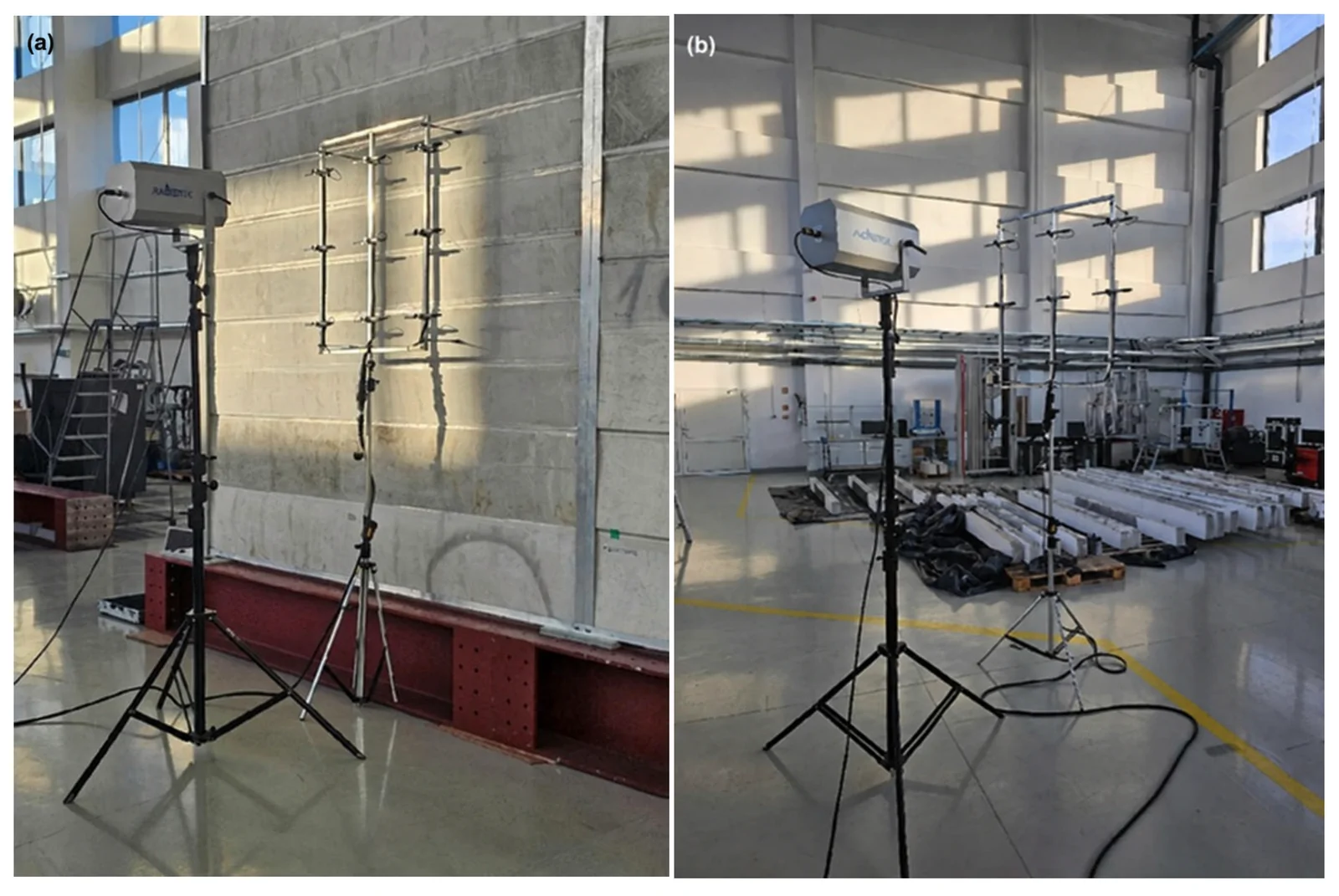
Measurement of the sound reflection coefficient: (**a**) view of the measurement setup and (**b**) reference impulse response measurement—measurement setup geometry identical to that with the screen, but without the screen between the microphone array and the speaker.

**Figure 6:**
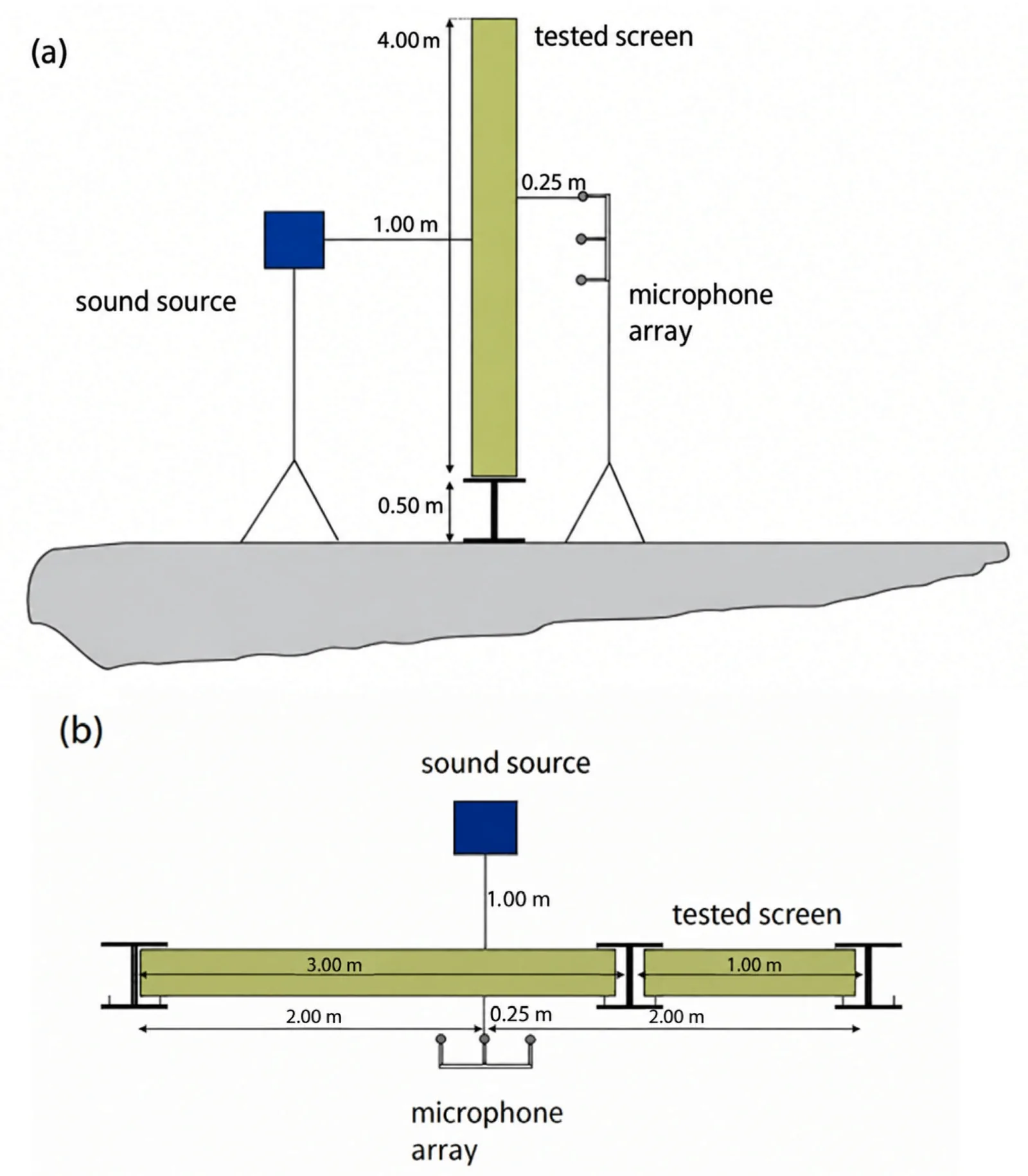
Measurement system: (**a**) side view and (**b**) top view.

**Figure 7:**
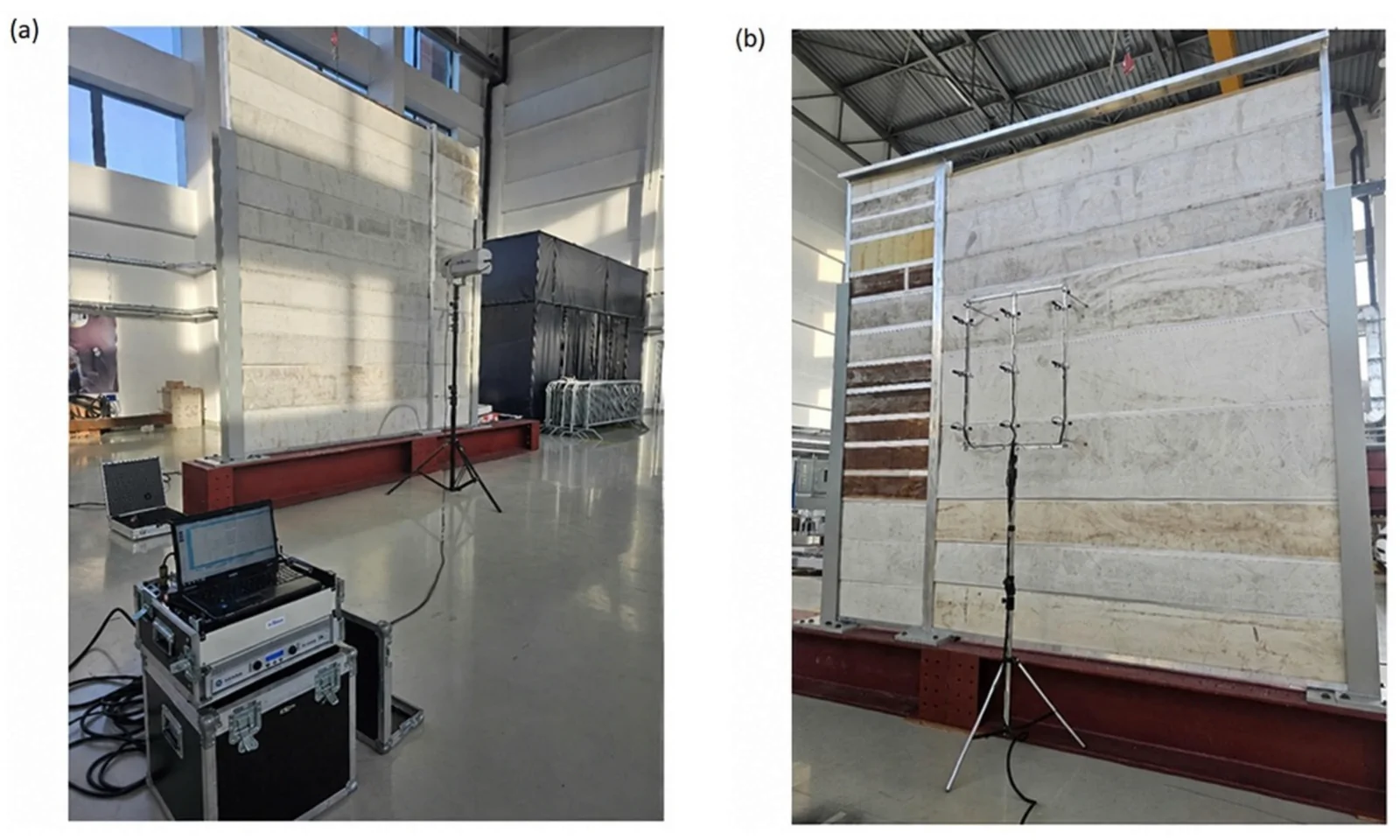
Performing the measurement: (**a**) view of the loudspeaker and (**b**) view of the microphone array.

**Figure 8:**
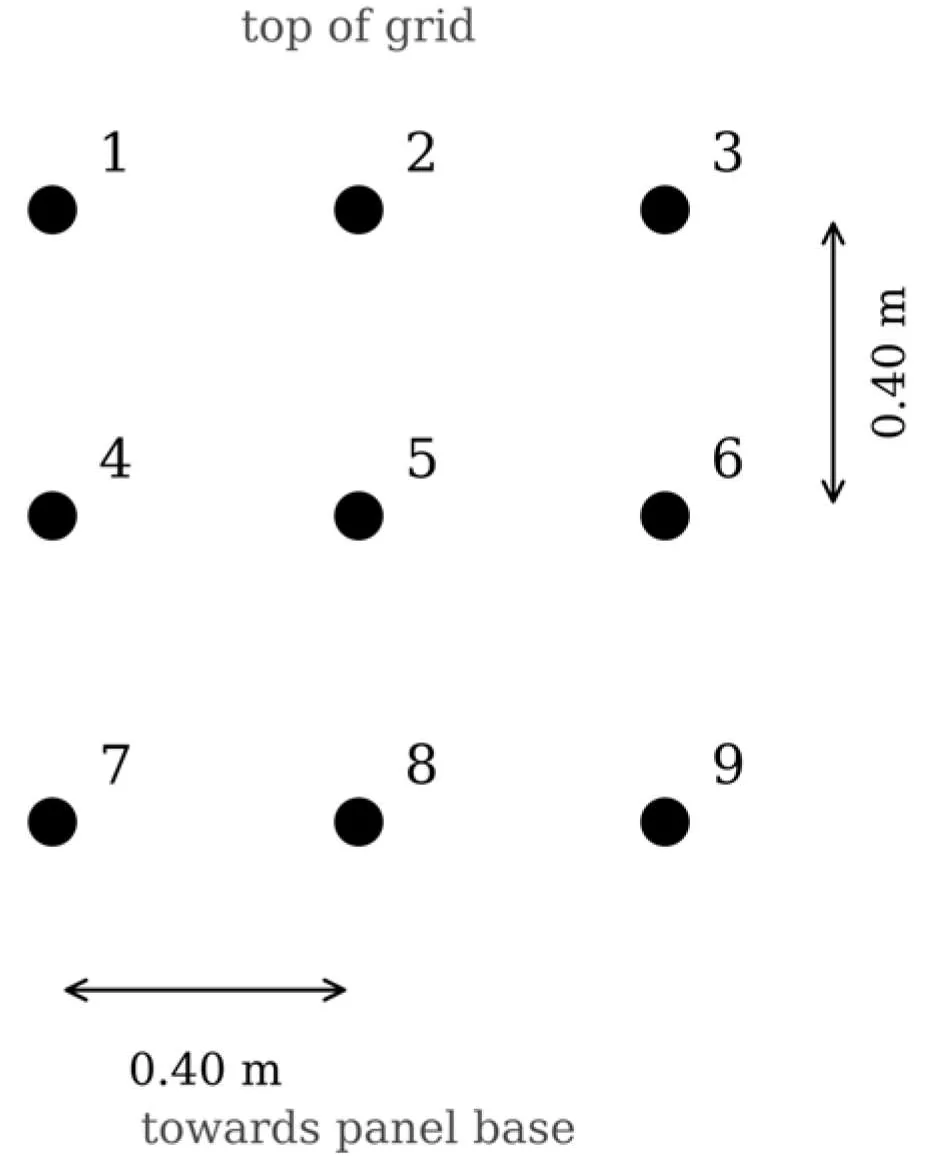
Arrangement of the nine microphone positions on the measurement grid, as specified in EN 1793-6 [7] (schematic drawn by the authors). Positions 7–9 form the lowest row, closest to the panel base.

**Figure 9:**
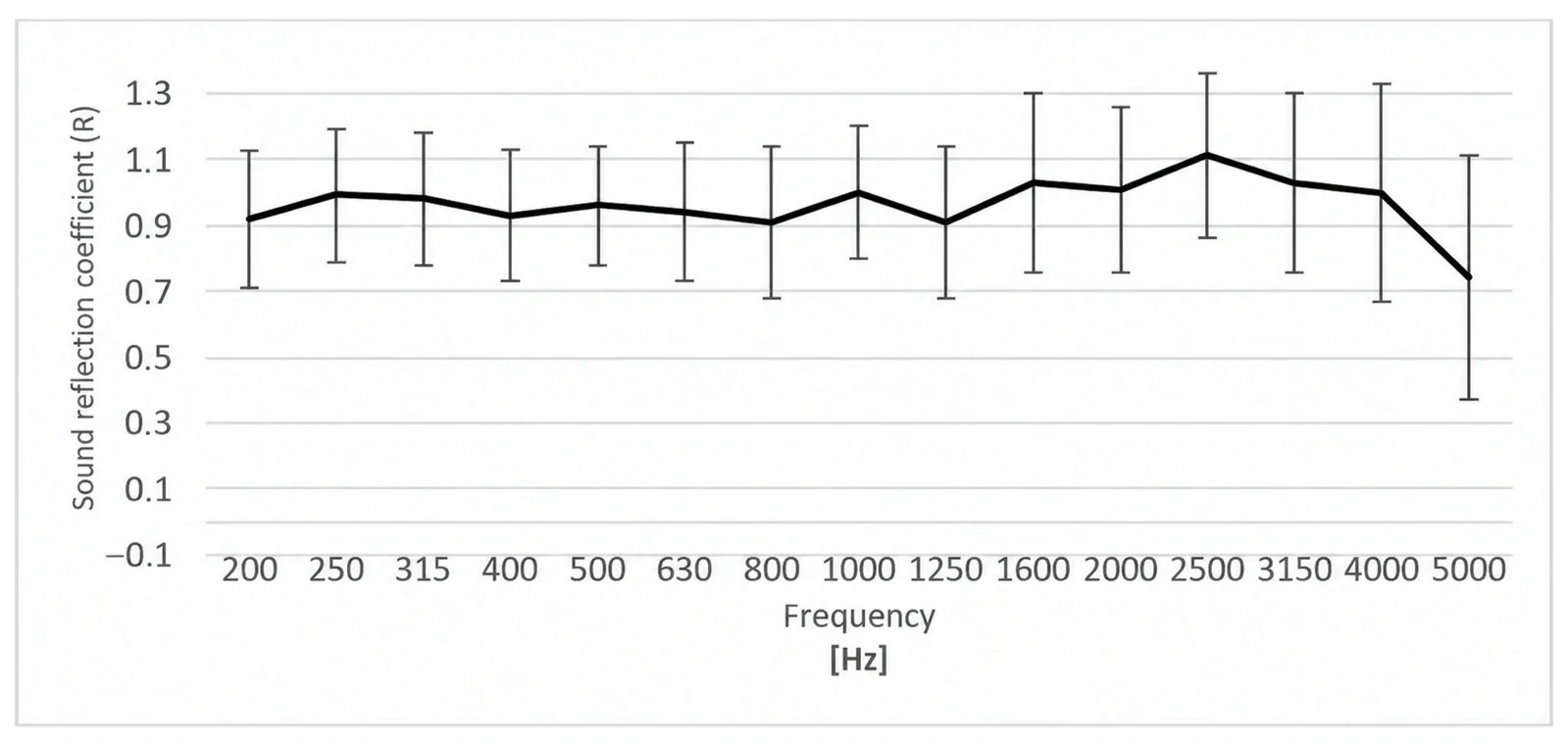
The graph of the sound reflection coefficient (*RI*) with uncertainties for one-third octave frequency bands in accordance with the standard EN 1793-5.

**Figure 10:**
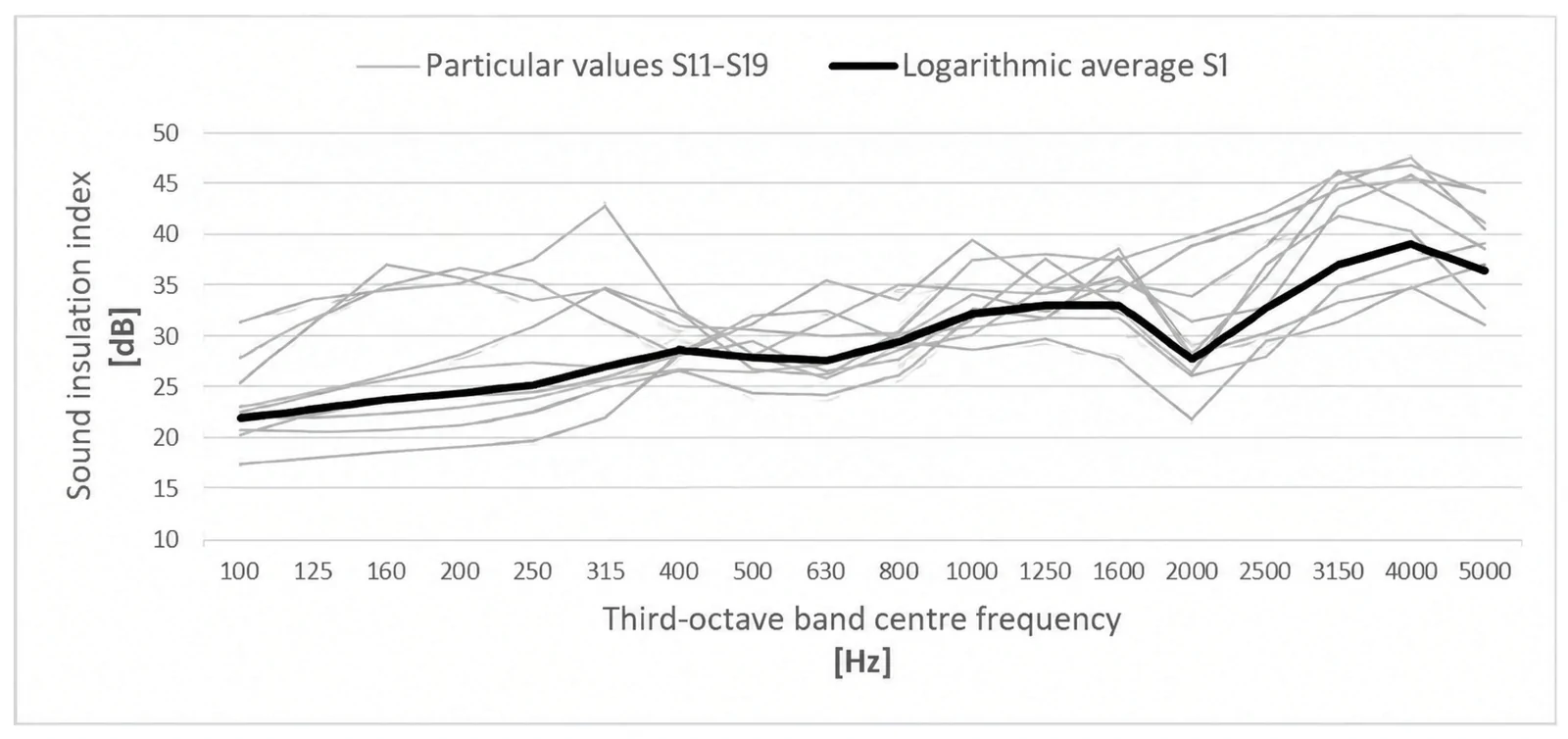
Graph of the sound insulation index *SI_E_* for one-third octave frequency bands in accordance with standard EN 1793-6.

**Figure 11:**
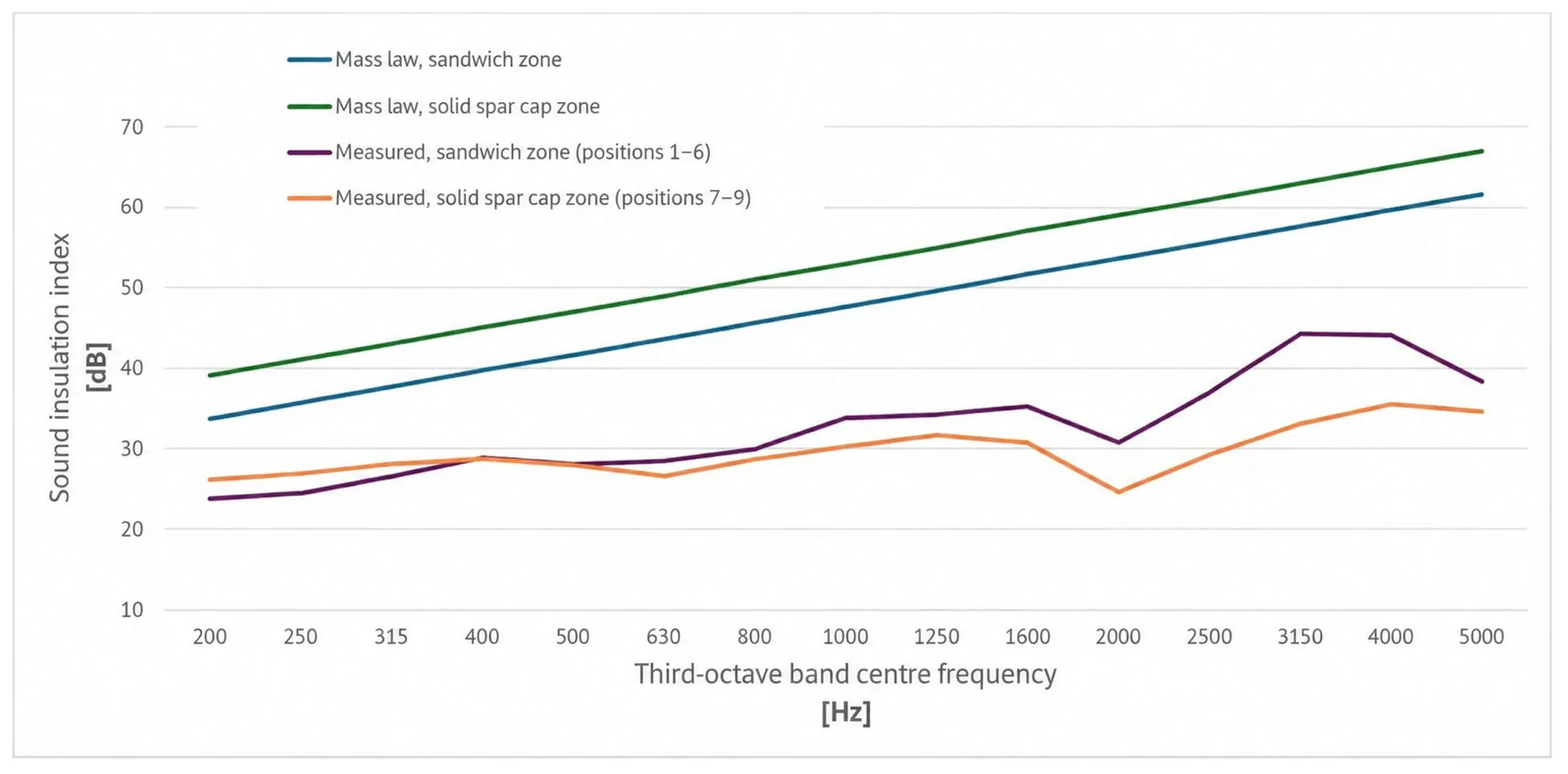
Measured sound insulation compared with the normal-incidence mass law, separately for each zone of the panel. Positions 1–6 face the sandwich zone (31.5 kg/m^2^); positions 7–9 face the solid spar cap zone (58.5 kg/m^2^).

**Figure 12:**
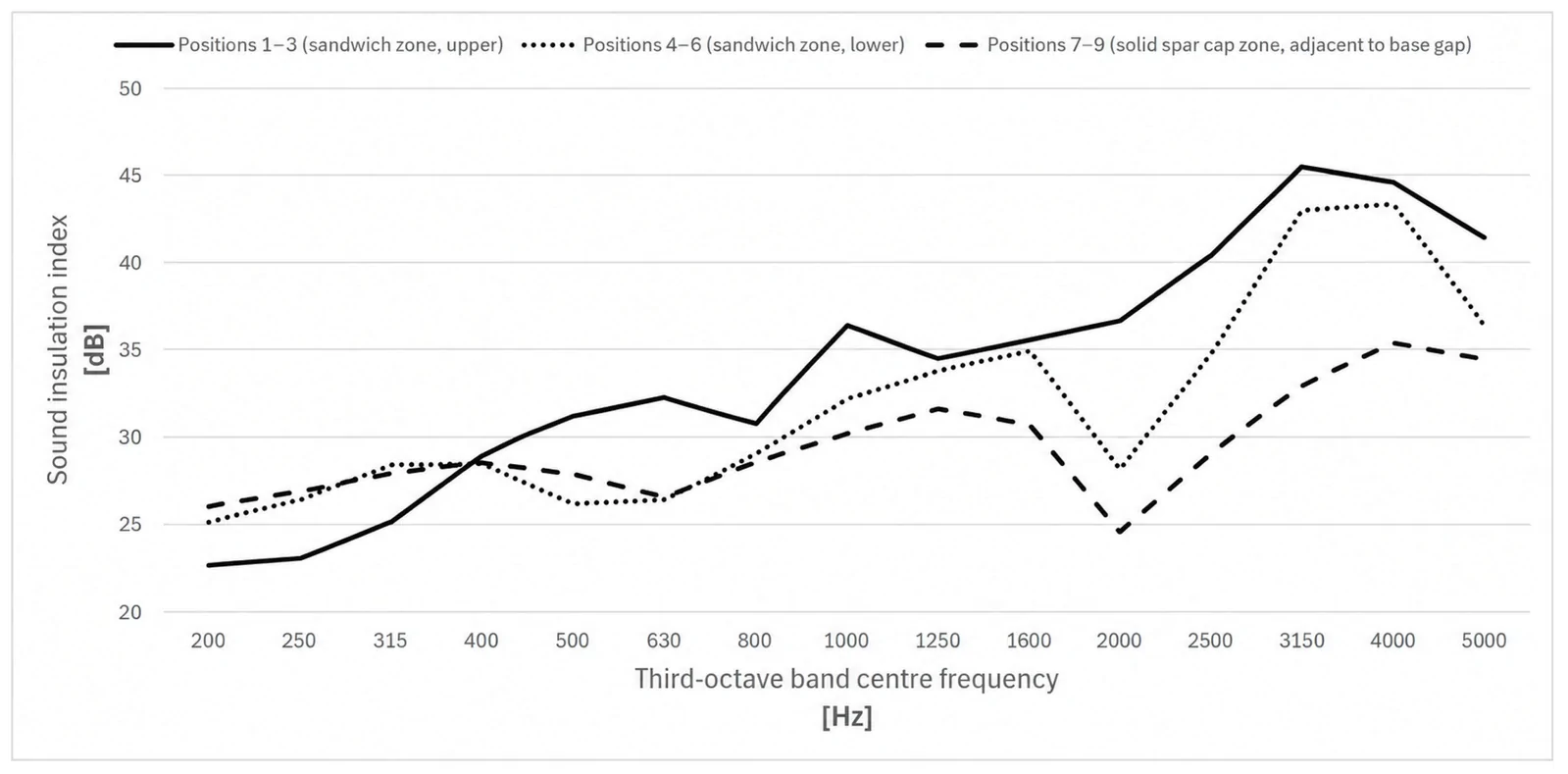
Sound insulation index vs. frequency per position group.

**Figure 13:**
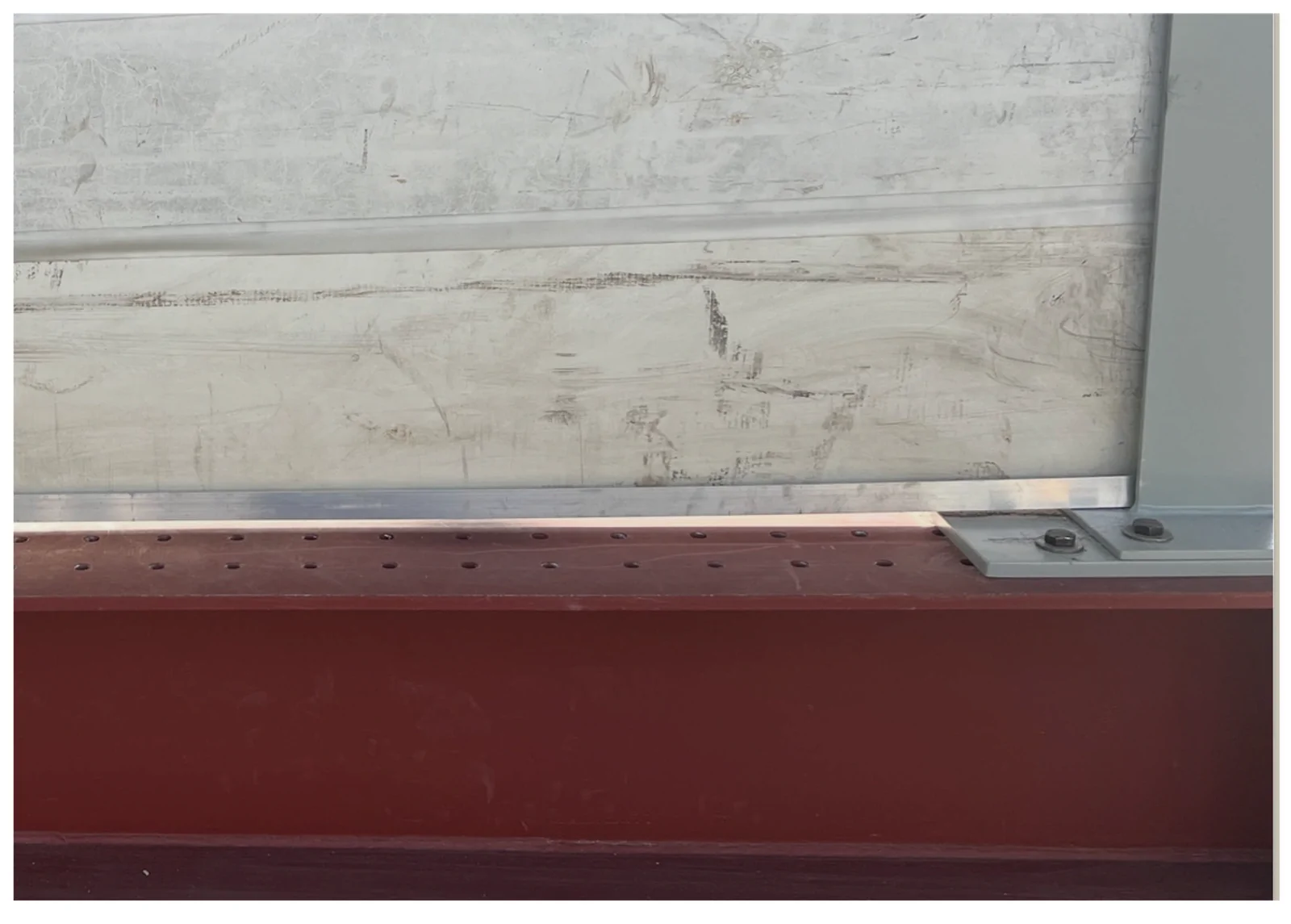
Gap between the steel stand and the panel frame.

**Figure 14:**
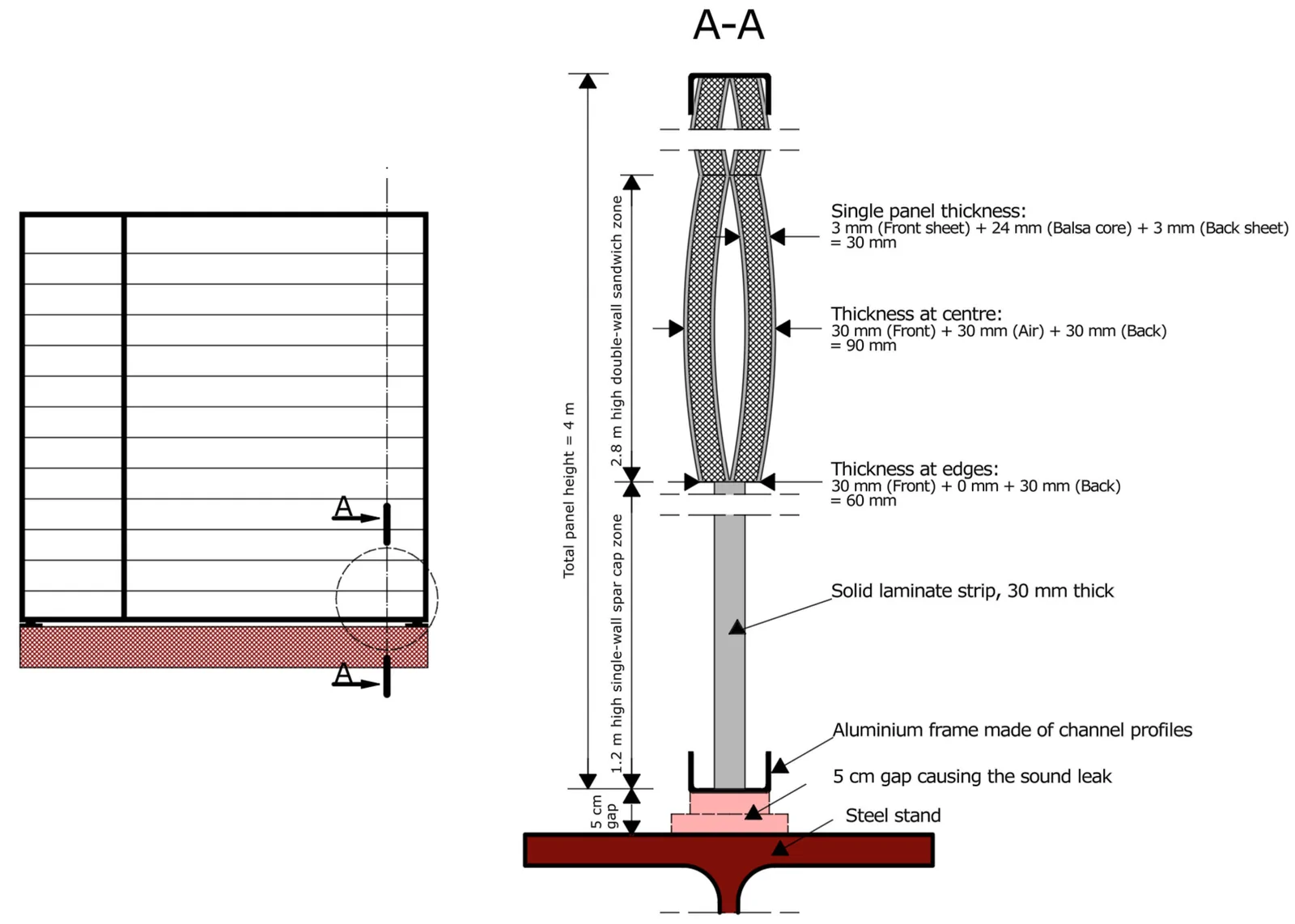
Schematic view of the noise barrier panel (**left**) with the cross-section location marked; detail of the cross-section (**right**).

**Table 1:** Mechanical properties of the laminates included in the turbine blade [4].

Properties	Type C Laminate	Type D Laminate (Sandwich)	
		Outer Layers	Core (Balsa)
Characteristic values			
Thickness—*t* [mm]	29.6	3.0 (each)	24.0
Young’s modulus in Z direction—*E_z_* [GPa]	24.8	5.9	(0.61–6.6)
Young’s modulus in Y direction—*E_y_* [GPa]	6.4	6.5	(0.013–0.098)
Shear modulus—*G_yz_* [GPa]	2.7	3.1	(0.04–0.36)
Density—ρ [kg/m^3^]	1976	1976	163
Compressive strength in Z direction—*Z_Ck_* [MPa]	344.2	217.7	-
Compressive strength in Y direction—*Y_Ck_* [MPa]	145.8	159.7	-
Tensile strength in Z direction—*Z_Tk_* [MPa]	405.8	88.5	-
Tensile strength in Y direction—*Y_Tk_* [MPa]	52.8	119.6	-
Shear strength—*S_yz_* [MPa]	26.4	44.2	(1.3–3.1)

**Table 2:** Measurement instrumentation and signal processing parameters.

Parameter	Specification
Signal processing	
Test signal	Maximum-length sequence (MLS)
Sequence length	32,768 samples
Number of averages	16
Sampling frequency	48 kHz
Bit resolution	24 bits
Anti-aliasing filter	Built-in to the analogue-to-digital converter of the audio interface
Adrienne window length	7.9 ms (positions 1–6); 5.9 ms (positions 7–9)
Frequency range	200 Hz–5 kHz (one-third octave bands)
Equipment	
Measurement interface	Behringer XR18 AIR (S/N: S191105508B/8), 18-channel
Microphones	9 × Behringer ECM-8000, ½″ condenser (S/N: 17280)
Calibrator	SONOPAN KA-50
Sound source	AKUSTIX SDS-2 (S/N: 1)
Amplifier	Crown XTi 1000 (S/N: 8001584192)
Signal generation and acquisition	GSW & 7dBm Electronics AB RoomCapture v. 1.67 software, Toshiba Satellite A660-10X computer

**Table 3:** Measurement results as single-number ratings.

${DL}_{RI}$	$U_{95}$
0.1	1.4

**Table 4:** Sound reflection index RI of the prototype in one-third octave bands, measured in this study (per-band values from the accredited test report [30] commissioned by the authors, unpublished).

F [Hz]	End Result with Uncertainty Included				
	${RI}_{1}$	${RI}_{2}$	${RI}_{3}$	RI	$U_{95}$
100 *	0.46	0.52	0.54	0.51	0.53
125 *	0.63	0.71	0.66	0.67	0.27
160 *	0.82	0.81	0.74	0.79	0.18
200	0.96	0.94	0.86	0.92	0.21
250	1.03	1.00	0.94	0.99	0.20
315	0.96	0.97	1.01	0.98	0.20
400	0.95	0.91	0.92	0.93	0.20
500	0.99	0.97	0.93	0.96	0.18
630	0.90	0.94	0.98	0.94	0.21
800	0.89	0.92	0.92	0.91	0.23
1000	1.05	0.98	0.97	1.00	0.20
1250	0.88	0.91	0.94	0.91	0.23
1600	1.10	1.02	0.96	1.03	0.27
2000	1.03	0.97	1.02	1.01	0.25
2500	1.30	1.05	0.98	1.11	0.25
3150	1.14	1.02	0.94	1.03	0.27
4000	1.00	0.99	1.01	1.00	0.33
5000	0.69	0.73	0.79	0.74	0.37
*DL_RI_* [dB]	0.03	0.18	0.20	0.1	1.4

* Below standard evaluation range, only for informational purpose.

**Table 5:** Sound insulation index SIE of the prototype at the nine microphone positions and the logarithmic average, measured in this study (per-band values from the accredited test report [31] commissioned by the authors, unpublished).

Third-Octave Band Centre Frequency [Hz]	Particular Values [dB]									Logarithmic Average
	${SI}_{1}$	${SI}_{2}$	${SI}_{3}$	${SI}_{4}$	${SI}_{5}$	${SI}_{6}$	${SI}_{7}$	${SI}_{8}$	${SI}_{9}$	SI
100 *	17.5	20.3	25.3	20.8	22.4	27.8	22.0	23.0	31.3	21.9
125 *	18.1	22.3	31.1	20.6	24.3	31.7	22.0	24.3	33.6	22.9
160 *	18.6	23.6	36.9	20.8	26.2	35.0	22.3	25.7	34.6	23.7
200	19.1	24.2	35.8	21.3	28.1	36.7	22.9	26.9	35.1	24.4
250	19.7	24.5	33.4	22.4	30.8	35.5	23.8	27.4	37.5	25.1
315	22.0	25.9	34.5	24.8	34.7	31.5	25.6	27.0	42.8	26.9
400	28.2	28.2	31.0	26.6	32.3	28.3	26.8	28.1	32.6	28.7
500	32.0	31.1	30.6	24.4	26.7	28.1	26.4	29.5	28.3	27.9
630	32.6	35.4	30.1	24.3	26.1	31.4	27.4	26.6	25.9	27.7
800	29.6	33.5	30.2	26.1	30.2	35.0	29.5	27.6	28.8	29.4
1000	34.1	39.4	37.4	31.9	30.8	34.6	28.6	32.7	30.1	32.2
1250	32.5	34.7	38.0	37.6	31.7	34.0	29.6	31.8	35.0	33.1
1600	35.3	34.3	37.5	32.7	37.7	35.8	27.6	31.8	38.5	33.1
2000	33.9	38.8	39.7	26.5	28.0	31.4	21.8	26.2	28.3	27.6
2500	38.7	41.2	42.1	36.9	35.8	32.8	29.4	28.0	30.3	32.6
3150	46.3	44.5	45.9	41.8	45.1	42.8	31.4	35.0	33.3	37.1
4000	42.7	45.5	46.8	40.3	47.5	45.8	34.8	37.2	34.7	39.1
5000	38.6	44.3	44.1	32.7	40.7	41.3	31.2	39.1	37.1	36.5

* Bands below 200 Hz are reported for information only and are excluded from the single-number rating, in accordance with clause 5.5.7 of [7].

**Table 6:** Single-number airborne sound insulation ratings and surface masses of road noise barriers of different materials.

Barrier Type	Typical *DL_SI,E_* (dB) *	Surface Mass (kg/m^2^)
Concrete [32]	26–40	180–240
Metal [32]	22–42	10–25
PMMA transparent [33]	26–43	15–25
Timber [23,24]	15–35	20–40
Reused GFRP (this study)	29.6	39.6

* Single-number ratings over the 200 Hz–5 kHz range. In situ ratings obtained under direct sound field conditions (*DL_SI_*, EN 1793-6) are quoted in preference to laboratory diffuse-field ratings (*DL_R_*, EN 1793-2) wherever both are available, since only the former are directly comparable with the present measurement. The two are related but not interchangeable, and laboratory values for a given material are commonly higher.

## Data Availability

The original contributions presented in this study are included in the article. Further inquiries can be directed to the corresponding author.

## Appendix A. Prediction of Coincidence Frequencies

The critical frequency of a solid, homogeneous panel, at which the bending wavelength matches the trace wavelength of incident sound, is classically estimated using thin-plate theory as [35]:

(A1) $$f_{c}=\frac{c^{2}}{2\pi }\cdot \sqrt{\frac{m_{s}}{D}},$$

where:

*c*—speed of sound in air, *c* = 343 m/s;

*m_s_*—surface mass density [kg/m^2^];

*D*—bending stiffness per unit width [N·m].

For the tested sandwich panel, the bending stiffness is governed by the face layers acting at a distance from the neutral axis. The tested panel consists of two bonded blade halves, forming a mostly symmetric cross-section with four GFRP faces (*t_f_* = 3 mm each) and two cores (*t_c_* = 24 mm each), resulting in a total thickness of 60 mm at the strip edges and 90 mm at mid-height (where the 30 mm air cavity opens). The bending stiffness varies along the strip height. At the edges (faces at d = ±28.5 mm and ±1.5 mm from the neutral axis), the stiffness is lowest; at the centre (faces at d = ±43.5 mm and ±16.5 mm), it is roughly 2.7 times higher. The edge geometry gives the lowest coincidence frequency and is used as the lower bound. Using the parallel axis theorem and neglecting the faces’ own moment of inertia:

(A2) $$D=\sum E_{f}\cdot t_{f}\cdot {d_{i}}^{2},$$

where:

$E_{f}$—face modulus;

$t_{f}$—face thickness;

$d_{i}$—distance from each face centroid to the panel’s neutral axis, $d_{1}=d_{4}=28.5\;\mathrm{mm},d_{2}=d_{3}=1.5\;\mathrm{mm}$.

Using the mechanical properties from Table 1 (*E_z_* = 5.9 GPa, *E_y_* = 6.5 GPa for the type D sandwich faces), the bending stiffness is *D_z_* = 28,800 N·m and *D_y_* = 31,800 N·m for the Z and Y directions, respectively. Inserting these values into the thin-plate formula yields lower-bound critical frequencies: *f_c,y_* = 590 Hz, *f_c,z_* = 619 Hz. These values apply to the sandwich zone, with local surface mass of 31.5 kg/m^2^.

In the solid cap zone, the section is a single laminate rather than a sandwich, and its bending stiffness per unit width follows from plate theory as:

(A3) $$D=\frac{E\cdot t^{3}}{12\;\left(1-\nu^{2}\right)},$$

with *t* = 29.6 mm and the moduli of the Type C laminate given in Table 1. Taking *ν* = 0.3, the bending stiffness is *D_z_* = 58,900 N·m and *D_y_* = 15,200 N·m and critical frequencies of 590 Hz and 1162 Hz respectively; taking *ν* = 0 shifts these to 619 Hz and 1218 Hz. The in-plane Poisson’s ratio of the cap laminate was not measured, but the resulting spread of about 30 Hz is small compared with the width of a one-third octave band at these frequencies and does not affect the interpretation. Table A1 presents the bending stiffness and predicted frequencies of the two laminate zones.

**Table A1:** Bending stiffness and predicted coincidence frequencies of the two laminate zones.

Zone	*m_s_* (kg/m^2^)	Direction	D (N·m) *	*f_c_* (Hz)
Sandwich, Type D, double wall	31.5	z	28,800	619
		y	31,800	590
Solid spar cap, Type C, single layer	58.5	z	58,900	590
		y	15,200	1162

* Values for the solid zone computed according to Equation (A3) with ν = 0.3. Taking ν = 0 gives 619 Hz and 1218 Hz.

Thin-plate theory overstates the dynamic bending stiffness of a thick sandwich with a compliant core. In thick sandwich structures, the transverse shear modulus of the core (*G_xz_* = 0.04–0.36 GPa for the balsa used here [25]) causes out-of-plane shear deformation that reduces the effective dynamic bending stiffness at higher frequencies. Consequently, as frequency increases, the bending wave speed no longer increases as f^1/2^ but asymptotically approaches the core shear wave speed:

(A4) $$c_{s}={\left(\frac{G_{xz}}{\rho_{c}}\right)}^{1/2}.$$

Applying the Kurtze-Watters shear correction

(A5) $$\frac{1}{{c_{B,eff}}^{2}}=\frac{1}{{c_{B,thin}}^{2}}+\frac{1}{{c_{s}}^{2}},$$

where *c_Beff_* is the effective (shear-corrected) bending wave speed, *c_B,thin_* = (ω^2^·D/m_s_)^0.25^ is the thin-plate bending wave speed, and c_s_ is the core shear wave speed shifts the predicted coincidence upward. The magnitude of the shift is poorly constrained by the available material data. The transverse shear modulus reported for this balsa spans 0.04–0.36 GPa, an order of magnitude, which places the shear-corrected coincidence anywhere between approximately 620 and 1190 Hz. Since the laminate is orthotropic, coincidence is spread over a frequency band rather than occurring at a single frequency [36]. The correction applies to the sandwich zone alone. The solid cap laminate has no compliant core, and its predicted coincidence is not shifted. The lower edge of the coincidence region, which is what the measurement resolves, is set by thin-plate theory and is unaffected.

Table A2 gives the thin-plate coincidence frequency of the sandwich zone for the plausible range of each input property. The lower edge is insensitive to the laminate parameters within those ranges: a ±20% change in face modulus moves it by about 12%, and changes in face thickness are largely self-cancelling because they raise the surface mass and the bending stiffness together. The dominant uncertainty is the transverse shear modulus of the balsa core, and it acts only on the upper extent of the coincidence region through the Kurtze–Watters correction, not on its lower edge. The wide shear-corrected range of 620–1190 Hz should therefore not be read as the uncertainty of the prediction being tested. What the measurement resolves is the lower edge at 590–620 Hz, which thin-plate theory fixes to within a few tens of hertz, and which is the feature compared with the plateau observed across the 500–800 Hz one-third octave bands.

**Table A2:** Sensitivity of the predicted thin-plate coincidence frequency of the sandwich zone (*f_c,z_*, nominally 619 Hz) to uncertainty in the input properties.

Parameter	Range Considered	Resulting *f_c,z_* (Hz)
Face modulus *E_f_*	5.9 GPa ± 20%	564–693
Face thickness *t_f_*	3.0 ± 0.5 mm	613–629
Core density *ρ_c_*	130–200 kg/m^3^	603–636
Core shear modulus *G*	0.04–0.36 GPa	620–1190
